# Targeting Insulin Signaling and TRAF2/JNK Pathway: A Comprehensive In Silico Study of *Uncaria tomentosa* Compounds

**DOI:** 10.3390/ijms27177724

**Published:** 2026-08-28

**Authors:** Bruna Freitas Marchi, Shraddha Parate, Vibhu Jha, Felipe Santiago Chambergo, Leif A. Eriksson, Viviane Abreu Nunes

**Affiliations:** 1 Department of Chemistry and Molecular Biology, University of Gothenburg (GU), 41390 Göteborg, Sweden; leif.eriksson@chem.gu.se; 2 Department of Life Sciences, Chalmers University of Technology, 41296 Göteborg, Sweden; 3 Institute of Cancer Therapeutics, School of Pharmacy and Medical Sciences, University of Bradford, Bradford BD7 1DP, UK; 4 Laboratory of Skin Physiology and Tissue Bioengineering, School of Arts, Sciences and Humanities, University of Sao Paulo (EACH-USP), São Paulo 03828-000, SP, Brazil

**Keywords:** unfolded protein response, insulin resistance, type 2 diabetes, *Uncaria tomentosa*, phytochemicals, in silico modeling

## Abstract

Type 2 diabetes (T2D) is a metabolic syndrome frequently associated with obesity and endoplasmic reticulum stress-mediated inflammation, which can trigger the unfolded protein response (UPR), impair insulin signaling, and promote apoptosis. To identify potential natural therapeutic candidates, this study investigated the mechanisms of action of 14 compounds from *Uncaria tomentosa* (UT), a medicinal plant from the Amazon rainforest, using in silico modeling. The study focused on the UPR, TRAF2/JNK pro-inflammatory signaling pathway, and insulin signaling pathways, which play key roles in T2D. Some of the UT compounds were docked against several human proteins involved in these pathways, and molecular dynamics simulations confirmed stable interactions between the target proteins (PERK, TRAF2, JNK, TNF-α, IRS-1, PI3K, AKT, GSK3β, and PPARγ) and four of the UT compounds, *5-Carboxystrictosidine*, *Cinchonain*, *Epicatechin* and *Mitraphylline*. Additionally, absorption, distribution, metabolism, excretion, and toxicity (ADMET) properties analyses were conducted to predict the four compounds, revealing suitable pharmacokinetic properties. These findings suggest that specific UT compounds may be used in experimental tests to whether investigate their therapeutic potential in managing T2D by modulating signaling pathways related to the conditions UPR, inflammation, and insulin resistance.

## 1. Introduction

Diabetes is a non-communicable disease that has been a large increase in cases in recent years [1]. The number of diagnosed cases of diabetes rose from 108 million in 1980 to 422 million in 2014, with a more rapid prevalence increase in low- and middle-income countries than in high-income countries [1,2]. More than 95% of diabetes cases are type 2 diabetes mellitus (T2D), a metabolic disorder with high mortality rates and significant healthcare expenses worldwide [1,3,4].

T2D is a chronic metabolic disorder characterized by hyperglycemia and impaired metabolic disturbances involving carbohydrate, lipid and protein metabolism, resulting from changes primarily associated with defects in insulin production, secretion, and/or action [3,5,6]. Insulin resistance, defined as an attenuated biological response to circulating insulin, is a central feature of both obesity and T2D [7].

Several molecular events contribute to insulin resistance, most of which are associated with oxidative stress, inflammation or endoplasmic reticulum (ER) stress [6,8,9]. ER stress is triggered when the protein folding and processing are misdirected, leading to activation of the Unfolded Protein Response (UPR), a cellular adaptive mechanism aimed at restoring proteostasis [9,10,11,12,13]. 

The UPR relies on three protein sensors, of which the inositol-requiring enzyme 1 (IRE1) is the most conserved. IRE1 consists of a luminal domain, a transmembrane helix, and cytosolic kinase and RNase domains. In its inactive state, the luminal domain binds the chaperone BIP, preventing its dimerization [9,14]. During the UPR, BIP dissociates, enabling dimerization, trans-autophosphorylation, and activation. Once IRE1 is activated, TNF receptor-associated factor 2 (TRAF2) is recruited to bind to this stressor sensor, mediating the activation of N-terminal c-Jun kinase (JNK) [14,15]. Particularly under the accumulation of ROS and misfolded proteins, several pathways are activated to protect cells from damage [9,14,16]. However, if the stress condition cannot be mitigated, the ER also signals molecular events involved in the expression of pro-inflammatory cytokines, such as tumor necrosis factor alpha (TNF-α) [14,17]. This cytokine induces TRAF2 and JNK expression, which regulate pro-apoptotic pathways to eliminate damaged cells [14,17].

The interaction between ER stress, inflammation and insulin signaling is particularly relevant to insulin resistance. Activated JNK also induces the phosphorylation of Ser312 (human numbering) in insulin receptor substrate 1 (IRS-1), thereby inhibiting its activity [18,19,20]. Consequently, it reduces the insulin-binding capability and also disrupts downstream signaling, further aggravating insulin resistance [9,19,21].

Given the complexity of these pathways, natural products have attracted interest as potential sources of molecules capable of simultaneously modulating multiple molecular targets in many diseases, including obesity and T2D [6,13]. Indeed, the World Health Organization (WHO) has proposed strategies to incorporate complementary and alternative therapies, such as phytotherapy, into the development of new technologies and innovations as global public health tools [1].

*Uncaria tomentosa* (UT), commonly known as “cat’s claw”, is a medicinal plant of the Rubiaceae family native to the Amazon rainforest [22]. Previous studies have already demonstrated antioxidant, anti-inflammatory, and immunomodulatory properties of UT extract, including attenuation of oxidative stress and ER stress-associated protein expression [6,13,23,24,25]. However, the specific compounds from the plant extract that interact with proteins involved in the UPR or insulin signaling pathways remain insufficiently characterized.

In this study, we used in silico modeling to explore the potential interactions of fourteen UT compounds with key target proteins, including PERK, TNF-α, TRAF2, JNK, IRS-1, PI3K, AKT, GSK3β and PPARγ.

The fourteen compounds included in this study were selected based on their consistent identification in phytochemical investigations of UT and their representation of the major classes of bioactive secondary metabolites reported for this medicinal species [22,25,26]. Rather than focusing on a single chemical family, the selection was designed to encompass the structural diversity of UT, including pentacyclic oxindole alkaloids (mitraphylline, isomitraphylline, pteropodine, isopteropodine, speciophylline, uncarine F), indole alkaloids (*5-carboxystrictosidine*), flavonoids (*epicatechin*), proanthocyanidins (*cinchonain*), and hydroxybenzoic acids (*7-deoxyloganate*) [25,26].

These compounds constitute the principal phytochemicals repeatedly isolated and chemically characterized from UT and are widely recognized as contributors to its reported anti-inflammatory, antioxidant, immunomodulatory and antidiabetic activities. Consequently, evaluating representative molecules from each major phytochemical class offers a comprehensive strategy for predicting and identifying the compounds most likely to interact with molecular targets involved in endoplasmic reticulum stress, inflammation, and insulin signaling.

## 2. Results

### 2.1. Molecular Docking of the UT Compounds

**Figure 1:**
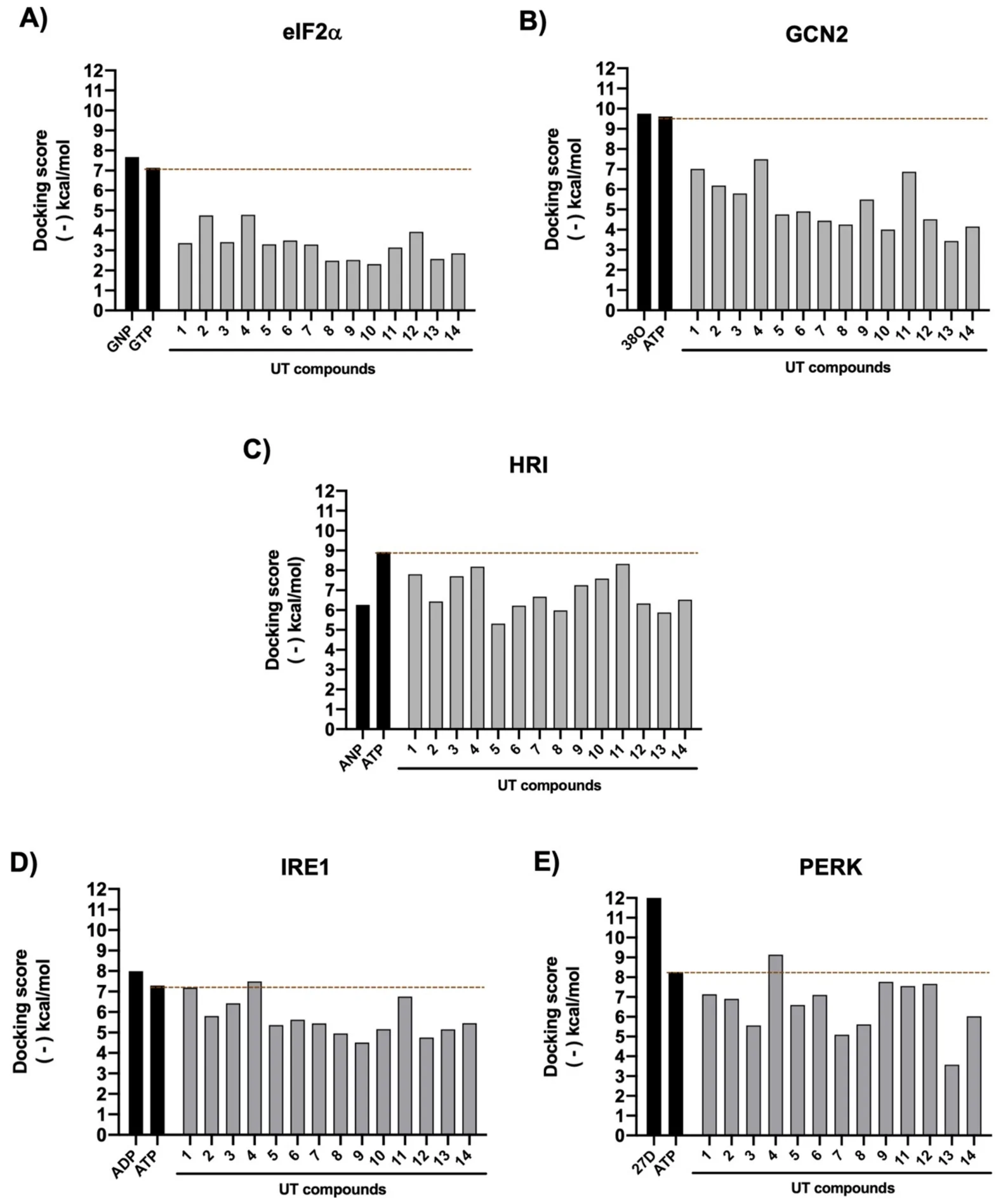
Affinity between the proteins (**A**) eIF2α, (**B**) GCN2, (**C**) HRI, (**D**) IRE1, (**E**) PERK, and the compounds present in the UT plant. Molecular docking scores between the fourteen UT compounds and the kinase binding site of the selected proteins, shown as negative values in kcal/mol. The natural substrates of each protein was used as a reference, and the corresponding docking score is indicated by the dashed line as a threshold for comparison with the UT compounds. Compound numbering is shown in Table 5 (Material and Methods).

**Figure 2:**
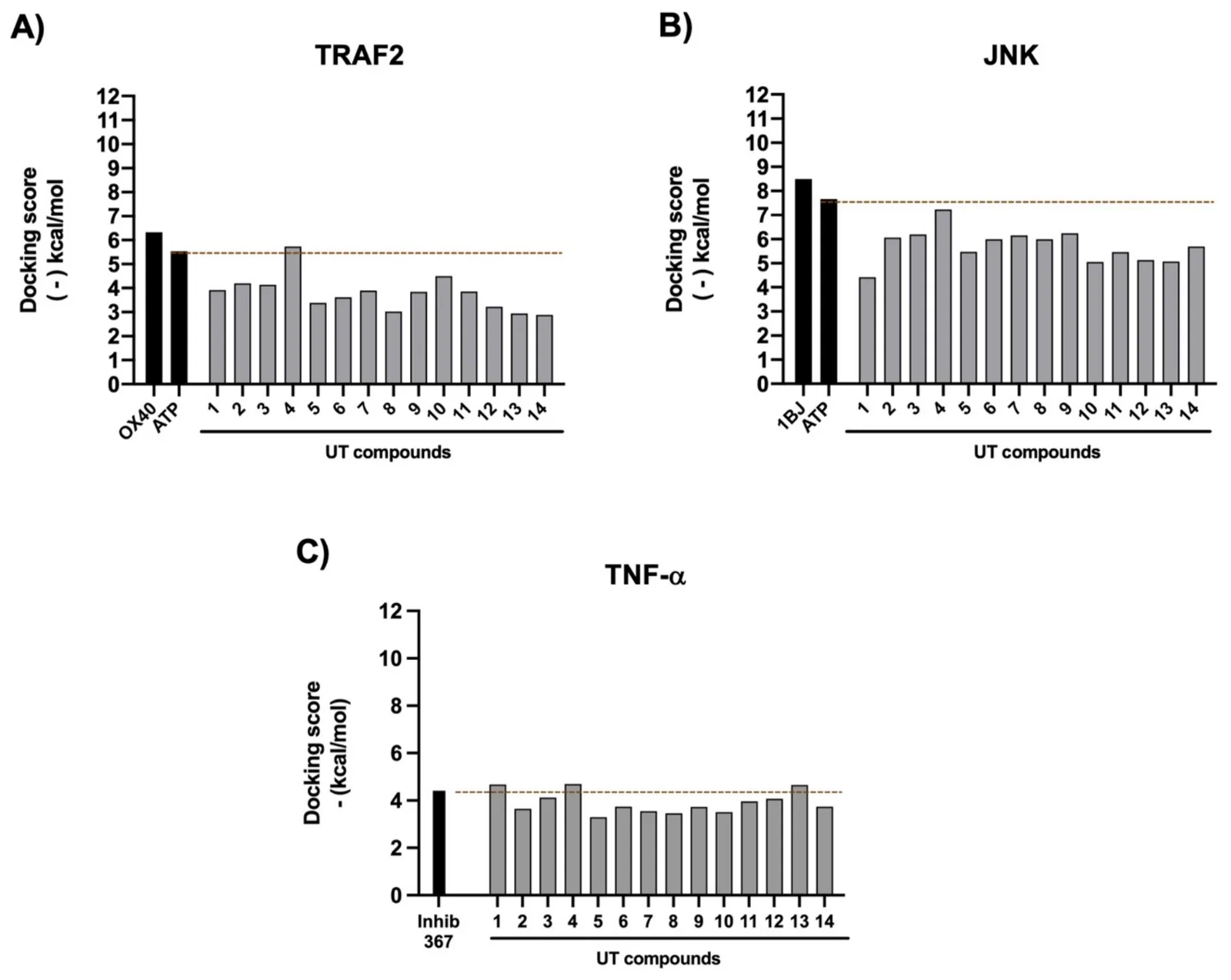
Affinity between the proteins (**A**) TRAF2, (**B**) JNK, (**C**) TNF-α and the UT compounds. Molecular docking score between the fourteen compounds and the binding site of the selected proteins, represented by negative values, in kcal/mol. The natural substrate or co-crystalized ligand of each protein was used as a reference, and the corresponding docking score is indicated by the dashed line as a threshold for comparison with the UT compounds. Compound numbering is shown in Table 5 (Material and Methods).

**Figure 3:**
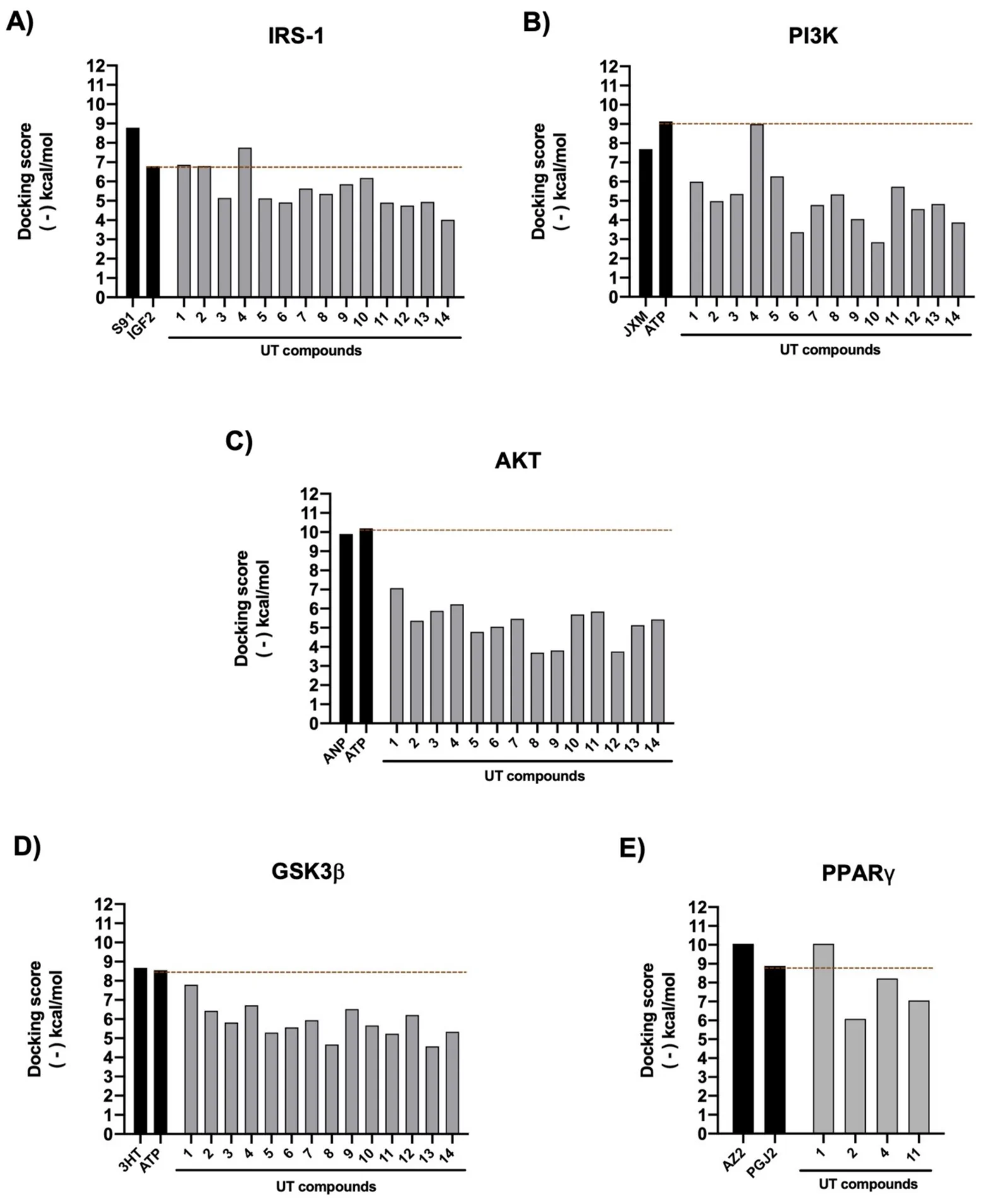
Affinity between the proteins (**A**) IRS-1, (**B**) PI3K, (**C**) AKT, (**D**) GSK3β, (**E**) PPARγ and UT compounds. Molecular docking score between the fourteen compounds and the binding sites of the selected proteins, represented by negative values, in kcal/mol. The natural substrates of each protein was used as a reference, and the corresponding docking score is indicated by the dashed line as a threshold for comparison with the UT compounds. Compound numbering is shown in Table 5 (Material and Methods).

Fourteen compounds present in the UT plant were preliminarily tested to evaluate their potential affinities with proteins involved in the ISR and TRAF2/JNK pathways, as shown in Figure 1, Figure 2 and Figure 3.

In the first group of proteins, the kinase eIF2α and the stress response receptors GCN2, HRI, IRE1, and PERK were analyzed (Figure 1A–E). Among the fourteen tested compounds, none exhibited higher affinity for the eIF2α than the substrate GTP, or higher affinity for GCN2, HRI, or IRE1 kinases than the substrate ATP. However, in the first group of proteins, *Epicatechin* (4) showed a significant interaction with PERK, with a binding energy of approximately −9.13 kcal/mol, which is significantly higher than that of ATP, as shown in Figure 1E.

In the second group of proteins, relating to the inflammation and cell survival pathway, TRAF2, JNK, and TNF-α were analyzed (Figure 2A–C). For TRAF2 (Figure 2A), ATP was bound at the inhibition site and showed an affinity of −5.53 kcal/mol. *Epicatechin* (4) showed affinity for the protein’s inhibition site, with a binding energy of −5.73 kcal/mol. In addition, in the JNK protein complex (Figure 2B), *Epicatechin* (4) also demonstrated strong interaction with the protein, with an affinity of −7.23 kcal/mol, in comparison to ATP binding with an affinity of −7.66 kcal/mol.

Finally, for the TNF-α cytokine (Figure 2C), the co-crystallized inhibitor 307, bound at the active site of the cytokine, displayed an affinity of −5.13 kcal/mol. For this system, *5-Carboxystrictosidine* (1), *Epicatechin* (4), and *Uncarine D* (13) demonstrated relative affinity for the residues of the same inhibition site, with binding energies of −4.68, −4.69, and −4.65 kcal/mol, respectively.

In the third group of proteins, relating to the insulin signaling pathway, IRS-1, PI3K, AKT, GSK3β and PPARγ were analyzed (Figure 3A–E). It was found that insulin-like growth factor 2 (IGF-2), bound to the phosphorylation complex of IR-IRS1 proteins that activate insulin signaling (Figure 3A), exhibited an affinity of −6.87 kcal/mol. The UT compounds *5-Carboxystrictosidine* (1), *7-Deoxyloganic acid* (2), and *Epicatechin* (4) showed affinities with residues at the same phosphorylation site of the complex, with binding energies of −6.86, −6,80 and −7.75 kcal/mol, respectively.

For the kinase PI3K (Figure 3B), the substrate ATP bound at the catalytic activity site, with an affinity of −9.13 kcal/mol, while *Epicatechin* (4) exhibited a relative affinity of −8.99 kcal/mol at the catalytic site of PI3K.

None of the compounds exhibited higher affinity for the kinases AKT and GSK3β than the substrate ATP (Figure 3C,D). For the PPARγ protein (Figure 3E), the prostaglandin J2 (PGJ2) bound at the protein active site, which activates PPARγ expression, showed an affinity of −8.88 kcal/mol. For this protein, only four of the 14 compounds were able to dock to the active site. In this case, *5-Carboxystrictosidine* (1) exhibited a strong affinity for the active site of PPARγ, with a binding energy of −10.05 kcal/mol (Figure 3E).

**Figure 4:**
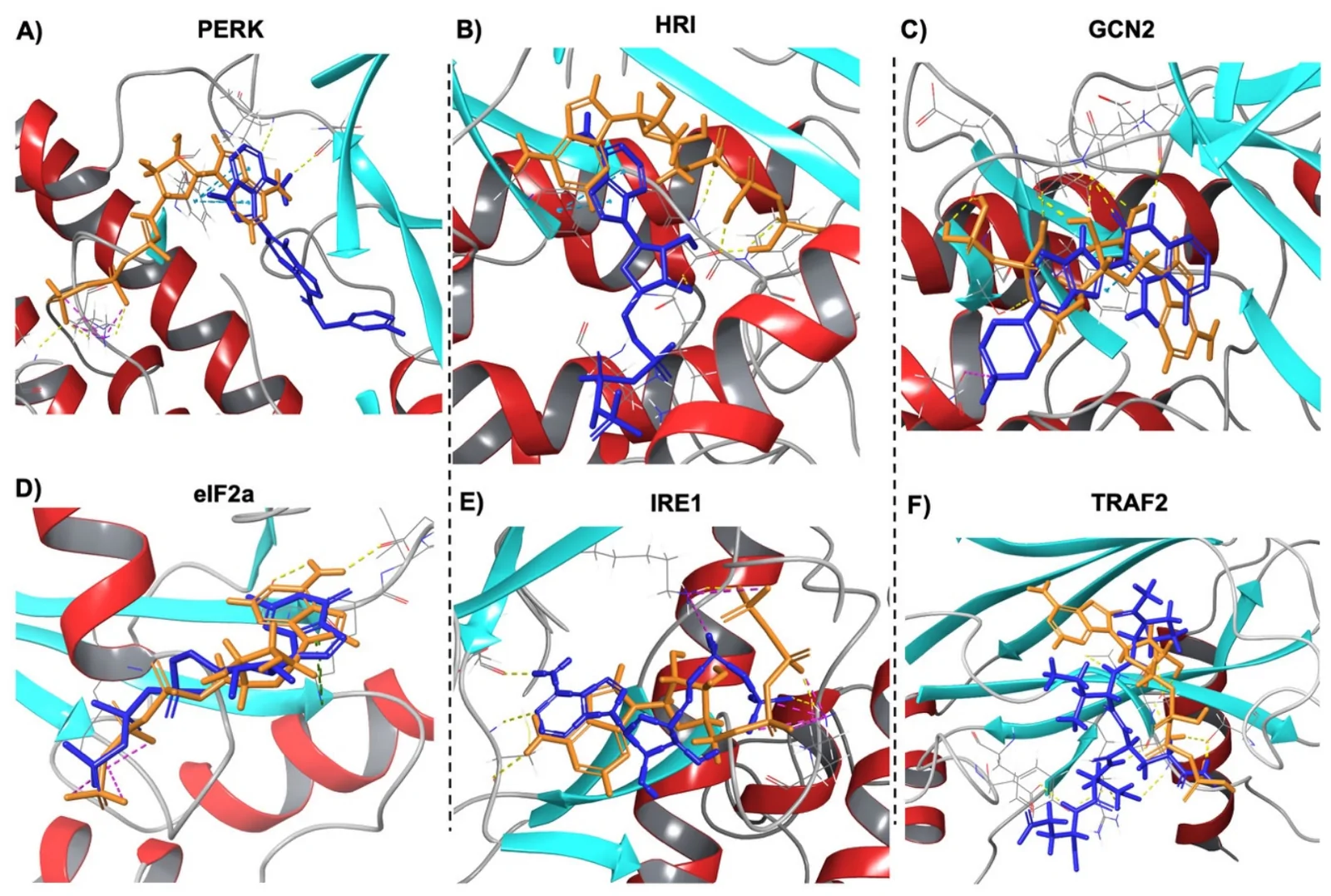
Superposition of the co-crystallized ligands (blue) and the corresponding substrates (orange) within the binding sites of proteins involved in the unfolded protein response (UPR). (**A**) PERK protein showing the superposition of the 27D ligand and ATP substrate; (**B**) HRI protein showing the superposition of the ANP ligand and ATP substrate; (**C**) GCN2 protein showing the superposition of the 38O ligand and ATP substrate; (**D**) eIF2α protein showing the superposition of the GNP ligand and GTP substrate; (**E**) IRE1 protein showing the superposition of the ADP ligand and ATP substrate; (**F**) TRAF2 protein showing the superposition of the OX40 ligand and ATP substrate. The overlap between the co-crystallized ligands and substrates highlights conservative substrate-binding pockets and supports the docking protocol by demonstrating that the predicted ligand-binding poses are consistent with experimentally resolved ligand orientations.

**Figure 5:**
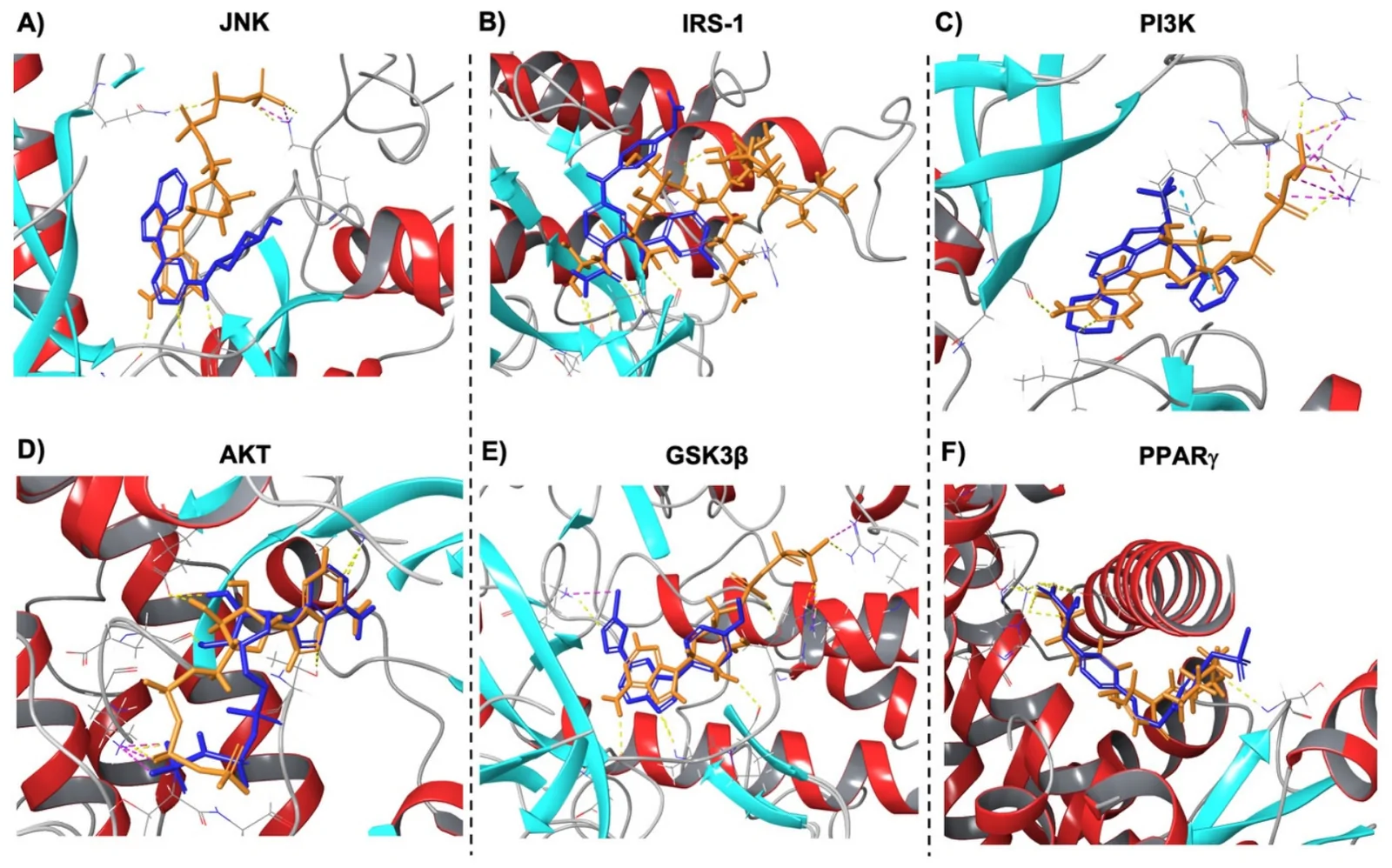
Superposition of the co-crystallized ligands (blue) and the corresponding substrates (orange) within the binding sites of proteins associated with insulin signaling and metabolic regulation. (**A**) JNK protein showing the superposition of the 1BJ ligand and ATP substrate; (**B**) IRS-1 showing the superposition of the S91 ligand and IGF2 substrate; (**C**) PI3K showing the superposition of the JXM ligand and ATP substrate; (**D**) AKT showing the superposition of the ANP ligand and ATP substrate; (**E**) GSK3β showing the superposition of the 3HT ligand and ATP substrate; and (**F**) PPARγ protein showing the superposition of the AZ2 ligand and PGJ2 substrate. The observed overlap between the co-crystallized ligands and the substrates indicates a conserved binding mode within the catalytic pockets, providing structural validation for the molecular docking methodology and supporting the reliability of the predicted ligand poses.

To evaluate the reliability of the molecular docking methodology, the binding poses of the co-crystallized ligands were superimposed with those of the corresponding endogenous substrates for each target protein (Figure 4 and Figure 5). In all analyzed proteins, the co-crystallized ligands exhibited a high similarity of spatial overlap with the native substrates within the respective binding pockets, indicating that the docking protocol accurately reproduced experimentally determined binding orientations.

For proteins involved in the unfolded protein response (Figure 4A–F), including PERK, HRI, GCN2, eIF2α, IRE1 and TRAF2, the co-crystallized ligands occupied the same catalytic or substrate-binding regions as ATP or GTP, demonstrating the conservation of the nucleotide-binding pockets across these proteins. The close superposition observed for each complex supports the structural integrity of the docking models and confirms that the predicted ligand poses are consistent with experimentally resolved conformations.

Similarly, proteins associated with insulin signaling and metabolic regulation (Figure 5A–F), including JNK, IRS-1, PI3K, AKT, GSK3β and PPARγ, showed substantial overlap between the co-crystallized ligands and their respective endogenous substrates (ATP, IGF-2 or PGJ2). This conserved binding pattern indicates that the active site was correctly identified during receptor preparation. These findings provide structural validation for the subsequent molecular docking analyses performed with the UT compounds and increase confidence in the predicted binding modes and affinity estimates reported for the selected targets.

### 2.2. MD Simulations

The stability of the binding between the UT compounds and target proteins was evaluated, along with the types of interactions established. For the molecular dynamics analysis of the first group, only PERK had adequate stability in interactions with *Epicatechin* (4) in the kinase binding site, with an average RMSD of 1.5 Å (Supplementary Figure S2). This result is in agreement with the docking study, showing that the complexes maintained RMSD values within 3.0 Å, indicating stable and consistent ligand–protein interactions.

In the second group of proteins, molecular dynamics simulations of UT compounds with TNF-α revealed that *Mitraphylline* (9) (Supplementary Figure S3) maintained stable interactions with the cytokine, with an RMSD of 3.0 Å. Furthermore, *Mitraphylline* (9) also displayed stable interactions with the TRAF2 inhibition site, with an RMSD of 2.7 Å (Supplementary Figure S4), compared to the results from ligand docking (Figure 2A).

On the other hand, TRAF2 and JNK showed binding affinity for *Epicatechin* (4), and molecular dynamics simulations confirmed that the interaction remained stable (Supplementary Figures S5 and S6), with RMSD values of 3.0 and 3.5 Å, respectively.

For the third group of proteins, molecular dynamics analysis demonstrated that *5-Carboxystrictosidine* (1) remained stable in the binding site of IRS-1 (Supplementary Figure S7), with an RMSD value of 2.5 Å. Additionally, the interaction between PI3K and *Epicatechin* (4) exhibited high stability with an RMSD of 1.5 Å (Supplementary Figure S8). On the other hand, the molecular dynamics of UT compounds with AKT and GSK3β revealed that *Cinchonain* (3) demonstrated partial stability in its interactions with the catalytic site of AKT (Supplementary Figure S9) and greater stability with the phosphorylation inhibition site of GSK3 (Supplementary Figure S10), with RMSDs of 5.5 and 2.5 Å, respectively, compared to the results observed in the ligand docking (Figure 3C,D).

Finally, the interaction of *5-Carboxystrictosidine* (1) with the active binding site of PPARγ was assessed (Figure 3E), given its role in enhancing insulin sensitivity in adipose tissue, skeletal muscle, and liver [27,28]. The compound was found to interact stably with PPARγ, with an RMSD of 2.0 Å (Supplementary Figure S11), in agreement with the docking studies.

During MD simulations, the main hydrogen-bonding and hydrophobic interactions were identified to determine their persistence under dynamic conditions, as summarized in Table 3. Representative interaction diagrams obtained from the MD trajectories are presented in the Supplementary Material (Figures S2–S11).

To further characterize the stability of the ligand–protein complexes, ligand flexibility was assessed using Root Mean Square Fluctuation (RMSF) analysis. At the same time, the relative binding affinity was estimated using MM/GBSA binding free energy calculations (Table 1). Overall, the ligands exhibited low RMSF values throughout the MD simulation trajectories, ranging from 0.9 to 2.7 Å, indicating limited conformational fluctuations and stable accommodation within the binding pockets during the simulations.

Among the analyzed complexes, Epicatechin–PERK exhibited the lowest ligand RMSF value of 0.9 Å, followed by *5-Carboxystrictosidine*–IRS-1 at 1.0 Å and *5-Carboxystrictosidine*–PPARγ at 1.1 Å, suggesting greater conformational stability of these ligands within their respective binding sites. Slightly higher fluctuations were observed for the *Epicatechin*–JNK complex with an RMSF value of 2.7 Å. However, the RMSF values remained within a range consistent with stable ligand binding throughout the simulation.

The MM/GBSA analysis yielded consistently negative free energy of binding values for all complexes, supporting favorable predicted ligand–protein interactions. The most favorable energies were observed for *5-Carboxystrictosidine*–IRS-1 (−72.68 kcal/mol), *Epicatechin*–PERK (−69.16 kcal/mol), *Cinchonain*–AKT (−66.44 kcal/mol), and *5-Carboxystrictosidine*–PPARγ (−61.37 kcal/mol). *Mitraphylline* also demonstrated favorable binding to TRAF2 (−51.94 kcal/mol), whereas the remaining complexes presented energies ranging from −35.82 to −58.46 kcal/mol, indicating stable predicted interactions across all investigated targets.

Taken together, these results, along with the RMSF and free energy of binding analyses, complement the RMSD results by demonstrating that the four selected UT compounds maintained relatively stable binding conformations throughout the simulations, while exhibiting favorable predicted binding free energies.

### 2.3. ADMET Profiling

Understanding the pharmacokinetics and safety profiles of compounds is crucial for assessing their absorption, distribution, metabolism, excretion, and toxicity (ADMET) properties, especially when evaluating their suitability as drugs.

ADMET analyses were conducted during the virtual screening stage for the four compounds, *5-Carboxystrictosidine*, *Cinchonain*, *Epicatechin,* and *Mitraphylline*, which showed the strongest interactions with target proteins of the stress response, TRAF2/JNK, or the insulin signaling pathway, as determined by the molecular dynamic tests.

**Figure 6:**
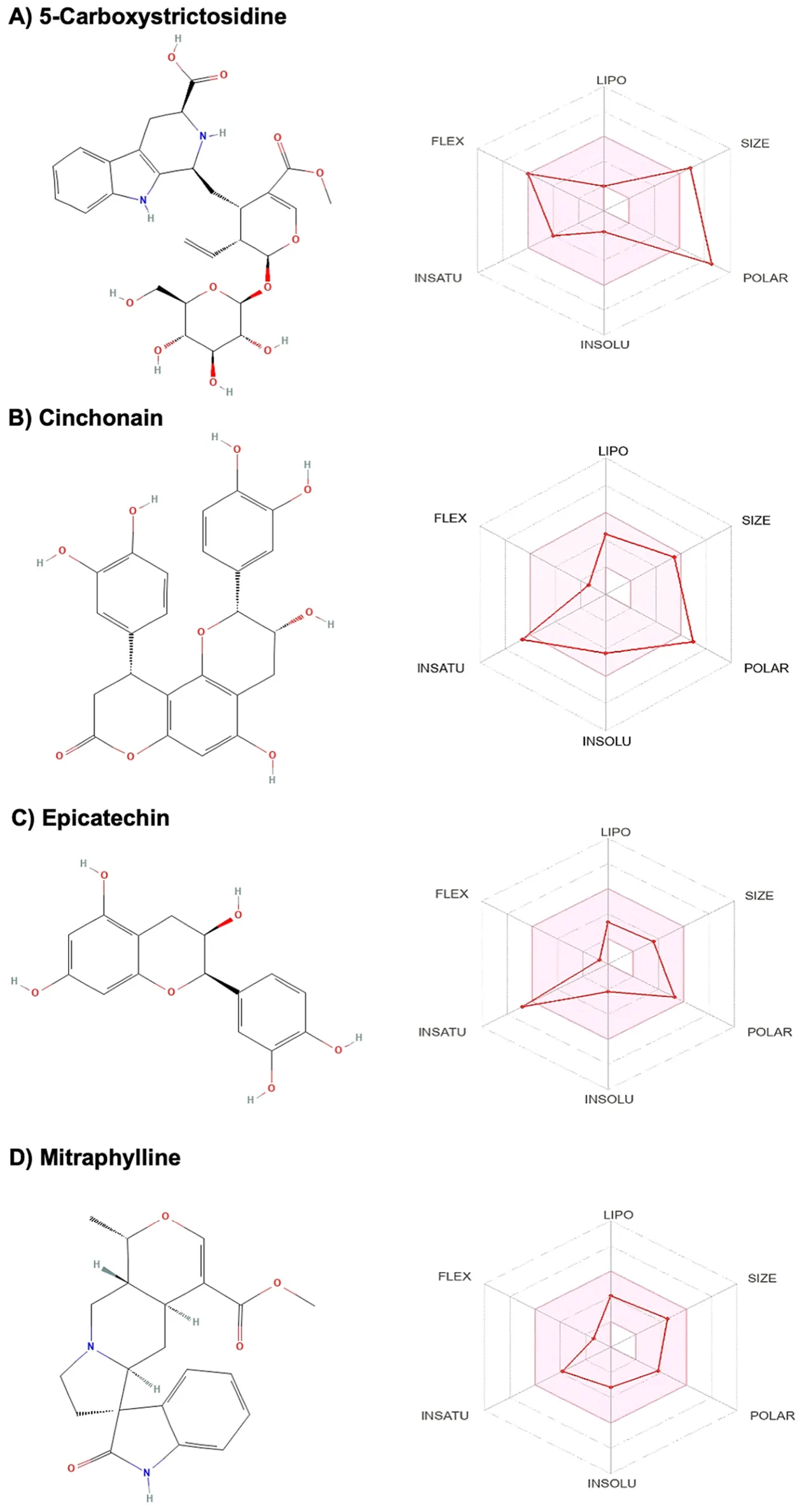
Chemical structures (left side) and main characteristics related to the oral bioavailability (right side). The four compounds, (**A**) *5-Carboxystrictosidine*; (**B**) *Cinchonain*; (**C**) *Epicatechin*; (**D**) *Mitraphylline*, were evaluated using bioavailability radar plots (pink area), represented by six physicochemical properties: lipophilicity (LIPO), molecular weight (SIZE), polar surface area (POLAR), solubility (INSOLU), saturation (INSATU), flexibility (FLEX).

The oral bioavailability graphs shown in Figure 6 illustrate the adjustments required to optimize each ligand’s behavior as a drug candidate (pink region of the radar plots). For *5-Carboxystrictosidine* (Figure 6A), adjustments in polarity and, to some extent, molecular size were indicated, suggesting that structural optimization could improve its pharmacokinetic profile. In the case of *Cinchonain* (Figure 6B), improvement in polarity was also highlighted, along with an increase in unsaturation, as suggested for *Epicatechin* (Figure 6C). Conversely, the radar plot for *Mitraphylline* (Figure 6D) demonstrated that it is structurally suitable, requiring only minor flexibility adjustments.

Overall, the ADMET analysis identified favorable pharmacokinetic characteristics together with important limitations that should be considered during future optimization.

In addition, some of the pharmacochemical ADMET properties were analyzed for the four selected compounds (Table 2). It was found that *5-Carboxystrictosidine* has strong hydrophilic properties, mainly due to its sugar moiety, leading to low absorption in the gastrointestinal tract (GIT). On the other hand, *Cinchonain*, *Epicatechin* and *Mitraphylline* have more lipophilic characteristics. Still, *Cinchonain* shows low predicted absorption by the GIT, which may restrict its oral bioavailability, while *Epicatechin* and *Mitraphylline* have high absorption by the GIT.

Most of the compounds, except *Cinchonain,* showed probabilities for affinity towards P-glycoproteins (P-gp). All of them present no significant risk of inhibiting the activity of the cytochrome P450 and isoenzymes CYP1A2, CYP2C19, CYP2C9, CYP2D6, CYP3A4, and CYP2E1. In addition, the four compounds did not present any predicted risk of toxicity to hepatocytes or other cells, except for cardiotoxicity, in which *5-Carboxystrictosidine* and *Mitraphylline* showed a moderate predicted risk of around 56–58% potential stimulation, indicating that additional toxicological evaluation could be required before considering therapeutic development.

## 3. Discussion

This study aimed to identify *Uncaria tomentosa* compounds [22,26] with potential interactions with key proteins involved in the unfolded protein response (UPR), inflammation, and insulin signaling, including PERK, IRS-1, PI3K, AKT, GSK3β, TNF-α, TRAF2, JNK, and PPARγ, using an integrated in silico approach.

The ligand docking was initially used to identify compounds with predicted affinities for the selected target proteins, relative to the corresponding natural substrates or co-crystallized ligands. MD simulations were subsequently used to assess the persistence and conformational behavior of selected ligand–protein complexes. Importantly, the computational results should be interpreted as evidence supporting the feasibility of the proposed binding modes rather than as direct biological activity or therapeutic efficacy.

In the first group of proteins, some of the transmembrane proteins involved in UPR, such as PERK and IRE1, and some that trigger ISR, such as HRI and GCN2, were evaluated [29]. No inhibitory effects were predicted between HRI, IRE1, or GCN2 and the 14 compounds. On the other hand, it was observed that the compound *Epicatechin* strongly binds to PERK kinase. This result suggests that the compound might interact with the kinase’s binding site sufficiently to remain stable [29]. Once PERK is activated, eIF2α is phosphorylated, which attenuates protein synthesis under ER stress conditions [30].

For eIF2α, no affinity was observed with the compounds, suggesting that the compounds present in UT do not directly attenuate protein synthesis [29,30]. This is further supported by the finding that PERK could also be inhibited by *Epicatechin*.

In the second group of proteins, the interactions of the UT compounds with the cytokine TNF-α, TRAF2 or JNK, expressed in one of the autophagy pathways, were evaluated [31,32].

During the ligand docking modeling, it was verified that the pro-inflammatory cytokine TNF-α exhibited affinity for three compounds: *5-Carboxystrictosidine*, *Epicatechin*, and *Uncarine D*. However, when evaluating the molecular dynamics of these compounds with the cytokine, no stable interactions with the binding site were found. This occurrence can be attributed to the fact that during molecular dynamics simulations, the ligand and protein can adopt different conformations, which may disrupt interactions initially predicted by docking. The dynamic behavior can reveal that some docking poses are not stable or energetically favorable under physiological conditions, leading to weaker or lost interactions during MD [29,33].

However, a second assessment by molecular dynamics showed that *Mitraphylline* interacts stably with TNF-α, suggesting that *Mitraphylline*, as an anti-inflammatory compound, could interfere with the activity of the pro-inflammatory cytokine TNF-α and subsequently modulate inflammatory responses [22,23].

Regarding the TRAF2 protein, while the flavonoid *Epicatechin*, known for its antioxidant action [34,35], demonstrated higher affinity with the binding site of TRAF2 than the natural substrate, *Mitraphylline* demonstrated the opposite (Figure 5A). However, after evaluating the dynamics between *Epicatechin* or *Mitraphylline* and TRAF2, stable interactions were observed between both compounds and the protein’s binding site.

From the docking, JNK showed a strong interaction with *Epicatechin*. When evaluating the dynamics between *Epicatechin* and the kinase, the interaction was also confirmed to be stable. These results suggest that *Epicatechin* and *Mitraphylline* can interact stably and potentially block the activity of TNF-α, TRAF2, and JNK, regulating oxidative stress and pro-inflammatory pathways [34,35].

To relate the results obtained for the UT compounds and to evaluate their potential to treat insulin resistance, the stability of the fourteen UT compounds was also assessed using molecular dynamics, focusing on the binding sites of target proteins in the insulin signaling pathway.

The compound *5-Carboxystrictosidine* showed stable binding to IRS-1 in both docking and MD simulations. The dynamics between PI3K and *Epicatechin* were also stable, even though a small difference was observed in the docking studies between *Epicatechin* (−8.99 kcal/mol) and ATP (−9.13 kcal/mol). This difference in affinity could be explained by additional interactions and conformational adjustments revealed in molecular dynamics simulations, which may compensate for differences in binding affinity suggested by limitations in docking scores [29,36].

Finally, when evaluating the interactions of the kinases AKT or GSK3β with the compounds, *Cinchonain* displayed stable binding to the kinases in the MD simulations.

Little is known about the relationship and interaction among *5-Carboxystrictosidine*, *Epicatechin*, and *Cinchonain* with the proteins of the insulin signaling pathway. However, according to some authors [22,23,26,34,37], these compounds, isolated from the plants *Uncaria tomentosa* or *Psychotria nuda*, have antioxidant characteristics and promote attenuation of ROS production in human erythrocytes, macrophages, and skeletal muscle tissue. This may be related to the attenuation of ER stress and the modulation of insulin receptor signaling [9,38].

Furthermore, the possible interaction between the transcription factor PPARγ and the compounds was evaluated. PPARγ antagonizes the metabolic syndrome of type 2 diabetes (T2D) by negatively regulating peripheral inflammatory processes, including the suppression of pro-inflammatory cytokines and increased insulin sensitivity in adipose tissue, skeletal muscle and liver [27,28,39,40]. When evaluating the affinity and binding stability between the UT compounds and PPARγ, it is suggested that this compound may act as a potential PPARy agonist, enhancing insulin sensitivity. However, the regulation of glucose and lipid metabolism [28,40], regarding *5-Carboxystrictosidine* also showed stable binding towards IRS-1 protein, a key mediator in the insulin signaling pathway, indicating its possible role in improving insulin receptor signaling and downstream metabolic effects [27,39,40].

The interactions of the four selected compounds with the target protein residues were further analyzed using MD simulations (Table 3).

The interactions found between *Epicatechin* and PERK residues Val651, Gln888, Phe955, Gly956 located in the DFG-*motif* and ATP binding site, correspond to 4 out of the 10 key residues found in the activation site (Leu598, Arg600, Gln888, Cys890, Lys938, Ser940, Asp954, Phe955, Gly956, Thr986) [41,42], which suggests that *Epicatechin* might impair the activation of the kinase and inhibit the expression under the UPR and stress responses.

The interactions of the same compound *Epicatechin*, a polyphenol known for its antioxidant role, and the JNK residues Glu109 and Met111 located in the inhibition site of the kinase, correspond to 2 out of 3 residues that mediate the suppression of JNK phosphorylation (Glu109, Leu110, Met111) [43,44,45], which suggests that *Epicatechin* might inhibit the activation of JNK, which plays a significant role in responses to oxidative stress [44].

Regarding the anti-inflammatory response of *Mitraphylline*, it was found to bind strongly to the residues Tyr59 and Tyr119, located in the inhibitory binding site of the pro-inflammatory cytokine TNF-α [46,47]. The binding of one or more compounds to this region alters the trimer symmetry and destabilizes the cytokine, thereby acting as TNFα inhibitors [46,47].

In relation to the insulin signaling pathway, Peasari et al. (2018) reported that IRS-1 residues Glu1077, Met1079, and Asp1083, which interacted strongly with *5-Carboxystrictosidine*, are located in the active site of this tyrosine kinase and modulate its insulin receptor activity [48]. Moreover, some authors [49,50] described that the residues Tyr670, Ile685, and Ser687, which are involved in the affinity between *Epicatechin* and the protein kinase PI3K, are located in the catalytic phosphorylation site and may contribute to the activation of the protein.

In addition, some authors [51,52,53] indicated that residues Leu158, Lys160, Glu236, Glu279, Thr436, and Phe439 in the AKT protein kinase, which exhibited affinity for *Cinchonain*, are located in the catalytic site of the kinase. For GSK3β, the residues Asp133, Gln185, and Asn186, which also interacted with the compound *Cinchonain*, are located in the active site of the protein kinase [54,55], suggesting that this compound may act as a modulator of GSK3β activity.

To determine whether UT compounds could also maintain stable binding conformations throughout lipotoxicity-induced insulin resistance target, their affinity for PPARγ was evaluated. Both His323 and Ser289 are among the residues that stabilize *5-Carboxystrictosidine* binding to the protein. According to some authors [56,57], interactions with residues His323 and Ser289 are characteristic of PPARγ agonists and contribute to the transcription factor, which is considered a target for hyperglycemia associated with T2D.

It is important to emphasize that molecular dynamics simulations evaluate the structural behavior of ligand–protein complexes within the computational model and cannot, by themselves, predict pharmacological efficacy. Therefore, the stability observed during the simulations should be interpreted as supporting the feasibility of the proposed binding mode rather than confirming biological activity.

Overall, *5-Carboxystrictosidine*, *Cinchonain*, *Epicatechin*, and *Mitraphylline* emerged as the four UT phytochemicals exhibiting the most favorable predicted interactions with the investigated target proteins. Although these compounds demonstrated promising binding characteristics, the ADMET analysis also identified important pharmacokinetic and safety limitations that should be considered during future lead optimization.

These findings emphasize that binding affinity alone is insufficient for compound prioritization, as favorable target interactions must be complemented by acceptable pharmacokinetic and toxicity profiles. Consequently, future medicinal chemistry efforts should focus on optimizing properties such as intestinal absorption, oral bioavailability, and safety, while preserving the predicted target affinity and key molecular interactions.

Therefore, this study serves as a base for conducting complementary experimental assays to validate and confirm the therapeutic potential of the predicted interactions, as well as to achieve a comprehensive understanding of the mechanism of action of the UT compounds.

## 4. Material and Methods

### 4.1. Selection of Compounds and Target Proteins

The crystal structures of 13 proteins involved in the UPR, ISR, or insulin signaling pathway in *Homo sapiens* were downloaded from the Protein Data Bank (www.rcsb.org) (Table 4). For the HRI kinase, due to the lack of a crystal structure, a homology model previously developed in the group using the YASARA software (version 24.10.5) was used [58].

In addition, the structures of fourteen compounds found in UT [22,25,26] were obtained from the PubChem database (https://pubchem.ncbi.nlm.nih.gov/, accessed on 23 August 2026) (Table 5). The chemical structures of the fourteen compounds are shown in Supplementary Figure S1. This diverse set of phytochemical structures enables comparison of different structural scaffolds against the selected protein targets, facilitating the identification of common and class-specific interaction profiles. This approach may also increase the likelihood of predicting and recognizing the phytochemicals that may contribute to the biological activity of UT, and provide a rational basis for prioritizing candidates for subsequent experimental validation.

Subsequently, the structures were transferred to Maestro software (version 2024-2) [59] for further preparation, docking, simulations, and analysis.

### 4.2. Protein Structure Preparation

All downloaded structures were prepared using the Protein Preparation Wizard tool in Maestro Schrödinger (version 2024-2) [59]. During preprocessing, hydrogen atoms and disulfide bonds were added to the initial coordinates. All water molecules located at a distance greater than 5.0 angstroms (Å) were deleted. To determine the likely protonation states of the side chains and the energy penalties associated with alternate protonation states, a pH of 7.0 ± 0.2 was applied.

The protein hydrogen bond assignments were then optimized in the H-bond Refine Tab using sample water orientations and PROPKA at pH = 7.0 [60]. For the initial restrained minimizations, the root-mean-square deviation (RMSD) for heavy atom convergence was set to 0.3 Å. The Optimized Potentials for Liquid Simulations (OPLS4) force field was employed, and hydrogen atoms were minimized while allowing sufficient heavy-atom motion to relax strained bonds, angles and clashes [61].

### 4.3. Compound Preparation and Molecular Docking

The structures of the compounds were prepared using the LigPrep module in Maestro. The Epik module [62] was used to evaluate possible ionization states at physiological pH 7.0 ± 0.2, and the OPLS4 force field was selected for the optimization.

**Figure 7:**
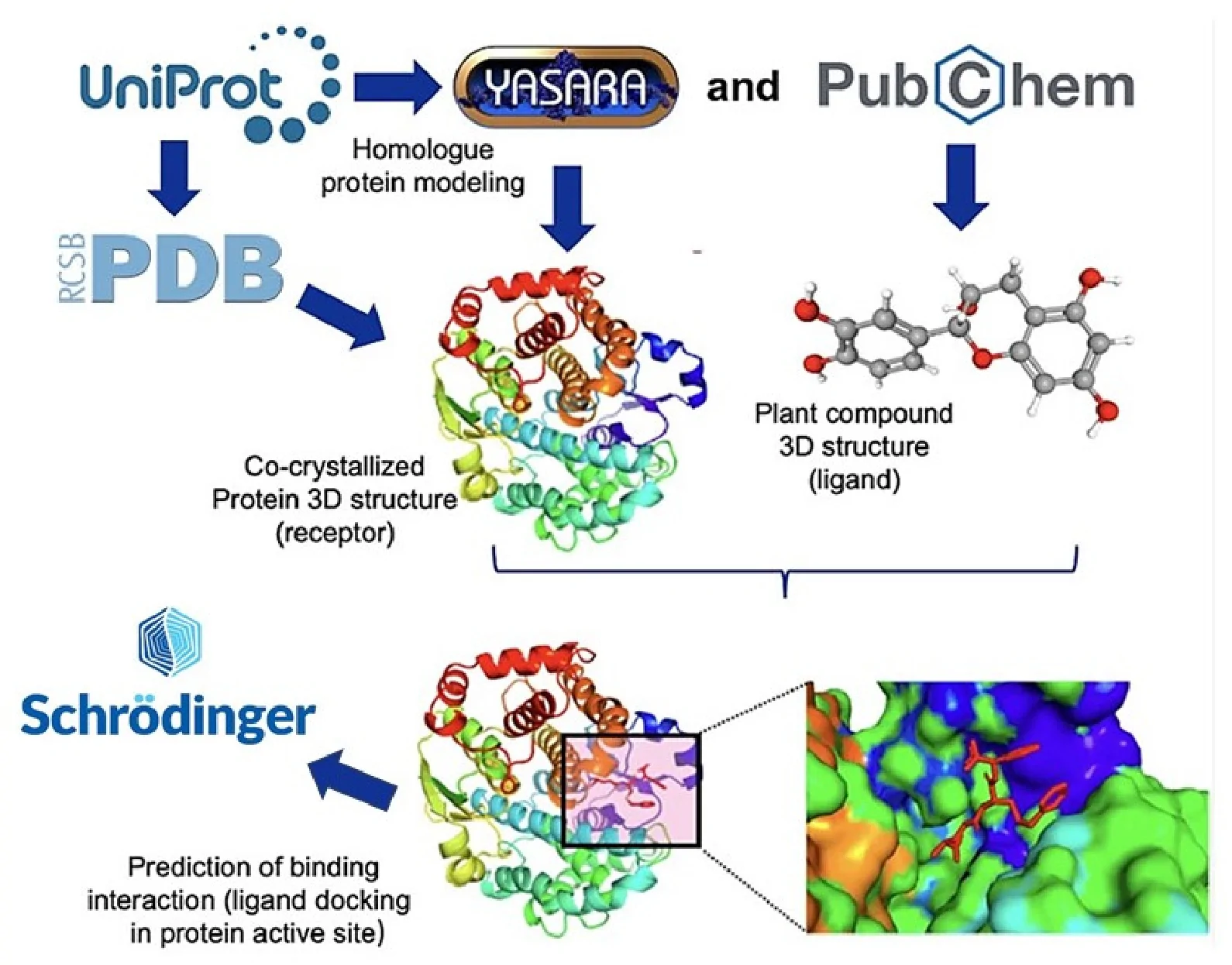
Schematic computational method of ligand docking, used to predict the binding affinity between the plant compounds and target proteins. Uniprot and PubChem databank were used to get the PDB of co-crystallized proteins (receptor) and UT compounds (ligands), respectively. YASARA software was used for homology protein modeling when needed [58]. After preparing each receptor and ligand, docking was performed using Glide in Maestro Schrödinger (version 2024-2) to predict binding interactions.

To perform molecular docking and predict interactions in protein–ligand complexes (Figure 7), the Induced Fit Docking (IFD) method in Glide Maestro Schrödinger (version 2024-2) was used [30,62]. The docking cubic grid was defined at the centroid of the bound co-crystallized ligand in the active binding site, with a side length of 20 Å.

To evaluate the reliability of the docking protocol, a redocking procedure was performed using the native substrates of each target protein (ATP, GTP, IGF2, and PGJ2) and the co-crystalized ligands. Each substrate was docked into the binding pocket corresponding to the position of the co-crystallized ligand (active site) in the reference protein structure. The predicted binding poses were subsequently compared with the experimentally characterized ligand-binding region to check the accuracy of the binding orientations and key interactions within the active site.

All fourteen ligands were docked into the active sites of the target proteins. During the initial docking procedure, the van der Waals scaling factor was set at 0.5 for both receptors and ligands. The Prime refinement step was applied to the side chains of residues within 5.0 Å of the ligand [63].

### 4.4. Molecular Dynamics (MD) Simulations

The stabilities of each protein-ligand complex were investigated through MD simulations employing the OPLS4 force field [61], using the Desmond package in Maestro Schrödinger software (version 2024-2) [59]. Equations of motion were integrated using the RESPA integrator with a 2-fs time step. Prior to the production run, each system underwent the default Desmond relaxation protocol, including energy minimization followed by restrained equilibration an NPT ensemble. The TIP3P force field was used to model water molecules, and periodic boundary conditions were applied with a 10 Å water buffer surrounding the protein within a cubic simulation box [59]. Na^+^ and Cl^−^ ions were added to neutralize the system and achieve a final NaCl concentration of 150 mM. Temperature and pressure were set to 300 k and 1 bar, using the Nose-Hoover thermostat and Martyna-Tobias-Klein barostat, respectively [64,65]. MD simulations were performed for 200–300 ns, sufficient for the ligand-protein complexes to reach relatively stable binding conformations and dynamic behavior. One independent MD simulation was initially conducted for each complex, followed by two additional replicate simulations to improve the statistical robustness and reproducibility of the results.

As a measure of protein mobility, the Root Mean Square Deviation (RMSD) of the α-carbons of the protein was calculated throughout the simulation, and the RMSD of the ligand heavy atoms during the trajectory of the simulation was also calculated, as a measure of ligand mobility [66]. The RMSD relates to the deviation of the ligand and protein α-carbon atoms from their initial suggested docking pose during the simulations.

In addition, the Root Mean Square Fluctuation (RMSF) of protein residues was analyzed to evaluate local flexibility and identify regions exhibiting higher conformational mobility throughout the simulation [66]. To further assess ligand binding, the Molecular Mechanics Generalized Born Surface Area (MM/GBSA) method was employed to estimate the free energy of binding in the protein–ligand complexes using representative frames extracted from the MD trajectories [29,62,63].

All data analyses, including RMSD calculations and protein–ligand contact analyses, were conducted using the Simulation Interaction Diagram (SID) tool in Maestro Schrödinger software (version 2024-2) [59].

The detailed ligand–protein interaction profiles, including hydrogen bonding and hydrophobic contacts observed throughout the MD simulations, are provided in Supplementary Figures S2–S11.

### 4.5. ADMET Profiling

To predict pharmacokinetic properties, SMILES (Simplified Molecular Input Line Entry Specification) codes of the selected compounds present in the UT plant were obtained and used to calculate absorption, distribution, metabolism, excretion, and toxicity (ADMET) properties using the SwissADME (www.swissadme.ch) and ProTox III (tox.charite.de/protox3/) servers.

The SwissADME platform enables submission of SMILES files for ligands and detects the structural fragments present in the compound. Descriptors corresponding to ADMET properties are calculated using internally implemented and validated models within the SwissADME tool. This platform incorporates several model libraries that analyze various ADMET property characteristics based on the physicochemical space of the molecule’s structural groups and the database information [36]. The results are provided as graphical outputs with legends indicating compliance or non-compliance with ADMET properties [36].

For toxic substructure analysis, the ProTox III server (version 3.0) was used. This tool evaluates structural alerts related to hepatotoxicity, mutagenicity, genotoxicity, carcinogenicity, cytotoxicity, cytochrome P450 interaction, and acute oral toxicity. The server’s libraries compile toxicological safety data (both in vitro and in vivo) from public databases and literature, with a total of 174 datasets/models. These models detect structural alerts in ligands by comparing the molecular information to known toxic groups and substructures of documented substances [48,54,67].

## 5. Conclusions

In summary, these findings provide a computational basis for prioritizing some of the phytochemicals present in UT herbal plant, for experimental investigation of their potential contribution to the biological effects within the molecular context of UPR, ER stress, insulin signaling regulation, insulin resistance and cell death. *5-Carboxystrictosidine*, *Cinchonain*, *Epicatechin* and *Mitraphylline* were found to exhibit the strongest affinities with the proteins involved in UPR and insulin resistance conditions, demonstrating significant interactions with key residues during the in silico modeling, although future experimental validations remain necessary.

## Figures and Tables

**Table 1:** Average ligand Root Mean Square Fluctuation (RMSF) and MM/GBSA binding free energy values calculated from the MD simulations of *5-Carboxystrictosidine*, *Epicatechin*, *Mitraphylline* and *Cinchonain* in complex with their respective target proteins (in blue). Ligand RMSF (Å) represents the average positional fluctuation of the ligand throughout the MD trajectory, with lower values indicating greater conformational stability within the binding pocket. The MM/GBSA free energy of binding (kcal/mol) corresponds to the average energy estimated from the MD trajectories, where more negative values indicate stronger predicted ligand–protein binding affinity. The reported values represent the average obtained over the simulation trajectories in two replicates.

UT Compounds	Protein	Average Values During the Trajectory	
		Ligand RMSF (Å)	Free Energy of Binding (kcal/mol)
*Epicatechin*	PERK	0.9	−69.16
	PI3K	1.4	−40.30
	TRAF2	2.0	−42.49
	JNK	2.7	−46.11
*Mitraphylline*	TNF-α	1.5	−35.82
	TRAF2	1.6	−51.94
*5-Carboxystrictosidine*	PPARγ	1.1	−61.37
	IRS-1	1.0	−72.68
*Cinchonain*	GSK3β	2.0	−58.46
	AKT	2.0	−66.44

**Table 2:** Pharmacochemical properties and toxicity profiles. Predicted ADMET properties of the four compounds *5-Carboxystrictosidine*, *Epicatechin*, *Mitraphylline*, and *Cinchonain* obtained through the SwissADME and Pro-Tox III servers, to indicate the physicochemical characteristics, lipophilicity, water solubility, pharmacokinetic aspects, similarity to other drugs (drug-likeness), and toxicity risk.

ADMET Properties		UT Compounds			
		*5-Carboxystrictosidine*	*Epicatechin*	*Mitraphylline*	*Cinchonain*
**Physicochemical characteristics**	MW (g/mol)	574.58 g/mol	290.27 g/mol	368.43 g/mol	452.41 g/mol
	Heavy atoms	41	21	27	33
	H+ acceptors	12	6	5	9
	H+ donors	7	5	1	6
**Lipophilicity**	Log P (−3 to 5)	−2.05	0.36	1.62	1.52
**Solubility in water**	Log S (ESOL, −4 to 0)	Very soluble (−1.68)	Soluble (−2.22)	Soluble (−3.18)	Moderately soluble (−4.33)
**Pharmacokinetics**	GTI absorption	Low	High	High	Low
	BBB permeability	No	No	No	No
	P-gp substrate	Yes	Yes	Yes	No
	CYP450 inhibitor and isoenzymes	No	No	No	No
**Drug-likeness**	Lipinski	No	Yes	Yes	Yes
**Toxicity risk**	Hepatotoxicity	No = 69%	No = 72%	No = 75%	No = 73%
	Cardiotoxicity	Yes = 56%	No = 99%	Yes = 58%	No = 79%
	Mutagenicity	No = 54%	No = 55%	No = 62%	No = 73%
	Cytotoxicity	No = 65%	No = 84%	No = 65%	No = 80%

**Table 3:** Characterization of the different protein interactions with the selected UT compounds (in blue) during the molecular dynamic simulations.

UT Compounds	Protein	Hydrogen Bond Interactions	Hydrophobic Interactions
		Amino Acids (Interactions ≥ 30%)	
*Epicatechin*	PERK	Val651; Lys621; Gln888; Arg891; Phe955; Gly956	no interaction
	PI3K	Tyr670; Asp761; Ile685; Gln683; Ser687; Glu692	Tyr670
	TRAF2	Ala48; Glu60; Leu73; Asp160; Phe161	no interaction
	JNK	Glu109; Met111	no interaction
*Mitraphylline*	TNF-α	--	Tyr59; Tyr119
	TRAF2	Met97; Asp160	no interaction
*5-Carboxystrictosidine*	PPARγ	His323; Ser289; Arg288	Leu340
	IRS-1	Gln1004; Glu1047; Glu1077; Met1079; Asp1083;	no interaction
*Cinchonain*	GSK3β	Asp133; Lys85; Gln185; Asn186	no interaction
	AKT	Leu158; Lys160; Ala232; Glu236; Glu279; Asp293; Thr436	Phe439

**Table 4:** Selected proteins and their respective codes in the Protein Data Bank (PDB) separated in three different groups (in blue), used in the in silico modeling. Group 1 involves proteins of the ISR and UPR receptor pathways; Group 2 involves proteins related to the inflammation and cell survival pathway; Group 3 involves proteins of the insulin signaling pathway.

	Target Proteins	PDB Code	Resolution (Å)
**Group 1**	Protein Kinase RNA-Like ER Kinase (PERK)	4M7I	2.34
	Inositol-requiring enzyme 1 (IRE1)	3P23	2.70
	Heme-regulated eIF2-α kinase (HRI)	Homology protein	-
	General control nonderepressible 2 (GCN2)	7QQ6	2.80
	Eukaryotic translation initiation factor 2 alpha and gamma (eIF2α)	8QZZ	3.35
**Group 2**	TNF Receptor Associated Factor 2 (TRAF2)	1D0A	2.0
	c-Jun N-terminal kinases (JNK)	4HYS	2.42
	Tumor necrosis factor alpha (TNF-α)	2AZ5	2.10
**Group 3**	Insulin receptor substrate (IRS-1)	2Z8C	3.25
	Phosphoinositide 3-kinase (PI3K)	4UWH	1.93
	Protein kinase B (PKB/AKT)	1O6L	1.60
	Glycogen synthase kinase 3 beta (GSK3β)	3F88	2.60
	Peroxisome proliferator-activated receptor gamma (PPARγ)	1I7I	2.35

**Table 5:** Selected compounds present in the UT plant, and their respective codes in the Pubchem databank.

	UT Compounds	PubChem Code
1	*5-Carboxystrictosidine*	CID: 44593370
2	*7-Deoxyloganic acid*	CID: 443322
3	*Cinchonain*	CID: 442675
4	*Epicatechin*	CID: 72276
5	*Hirsuteine*	CID: 3037151
6	*Hirsutine*	CID: 3037884
7	*Isomitraphylline*	CID: 11726520
8	*Isorhynchophylline*	CID: 3037048
9	*Mitraphylline*	CID: 94160
10	*Rhynchophylline*	CID: 5281408
11	*Strictosamide*	CID: 10345799
12	*Uncarine C (Pteropodine)*	CID: 10429112
13	*Uncarine D (Speciophylline)*	CID: 168985
14	*Uncarine E (Isopteropodine)*	CID: 9885603

## Data Availability

Protein structures after preparation, docked structures of the target proteins and UT compounds, plus the MD simulations, are freely available for download at zenodo.org (https://zenodo.org/records/17458126, accessed on 27 August 2026).

## Supplementary Materials

The following supporting information can be downloaded at: https://www.mdpi.com/article/10.3390/ijms27177724/s1.
