# Supplementary material for: Targeting Insulin Signaling and TRAF2/JNK Pathway: A Comprehensive In Silico Study of Uncaria tomentosa Compounds
Source: Int J Mol Sci. 2026 Aug 28;27(17):7724. doi: 10.3390/ijms27177724 (PMC13566028; doi:10.3390/ijms27177724)
Supplement: Supplementary file 1 [file ijms-27-07724-s001.zip › ijms-4422442-supplementary.pdf]

Targeting insulin signaling and TRAF2/JNK pathway: a comprehensive *in silico* study of *Uncaria tomentosa* compounds

Bruna Leticia de Freitas-Marchi [1] *, Shraddha Parate [2], Vibhu Jha [3], Felipe Santiago Chambergo Alcalde [4], Leif A. Eriksson [1], Viviane Abreu Nunes [4] *

[1] Department of Chemistry and Molecular Biology, University of Gothenburg (GU), Göteborg, Sweden;

[2] Department of Life Sciences, Chalmers University of Technology, Göteborg, Sweden;

[3] Institute of Cancer Therapeutics, School of Pharmacy and Medical Sciences, University of Bradford, United Kingdom;

[4] Laboratory of Skin Physiology and Tissue Bioengineering, School of Arts, Sciences and Humanities, University of Sao Paulo (EACH-USP), São Paulo, SP, Brazil

* Corresponding authors:

Viviane Abreu Nunes / Bruna Leticia de Freitas Marchi

Email: vanunes@ib.usp.br / bruna.marchi@gu.se

SUPPLEMENTARY MATERIAL

5-carboxystrictosidine

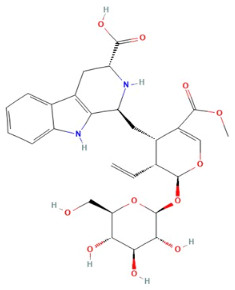

7-deoxyloganic acid

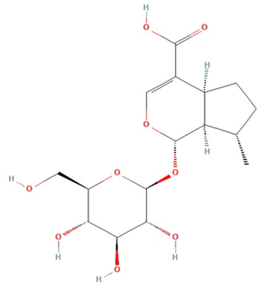

Cinchonain

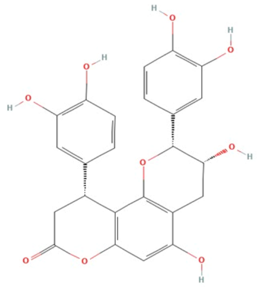

Epicatechin

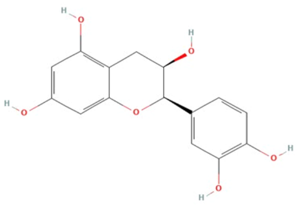

Hirsuteine

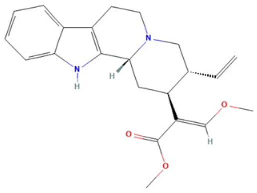

Hirsutine

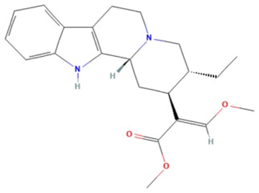

Isomitraphylline

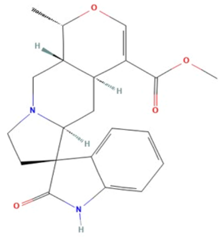

Isorhynchophylline

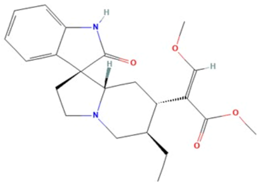

Mitraphylline

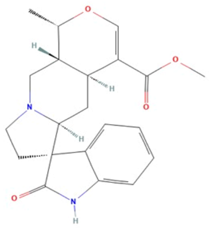

Rhynchophylline

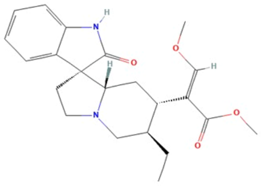

Strictosamide

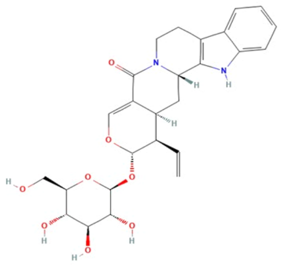

Uncarine C (Pteropodine)

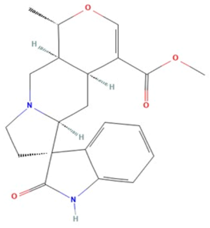

Uncarine D (Speciophylline)

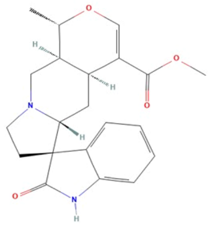

Uncarine E (Isopteropodine)

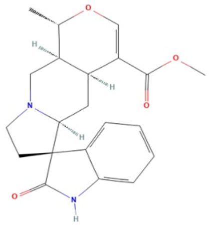

Figure S1. Chemical structures of the 14 compounds found in UT, obtained from the PubChem database

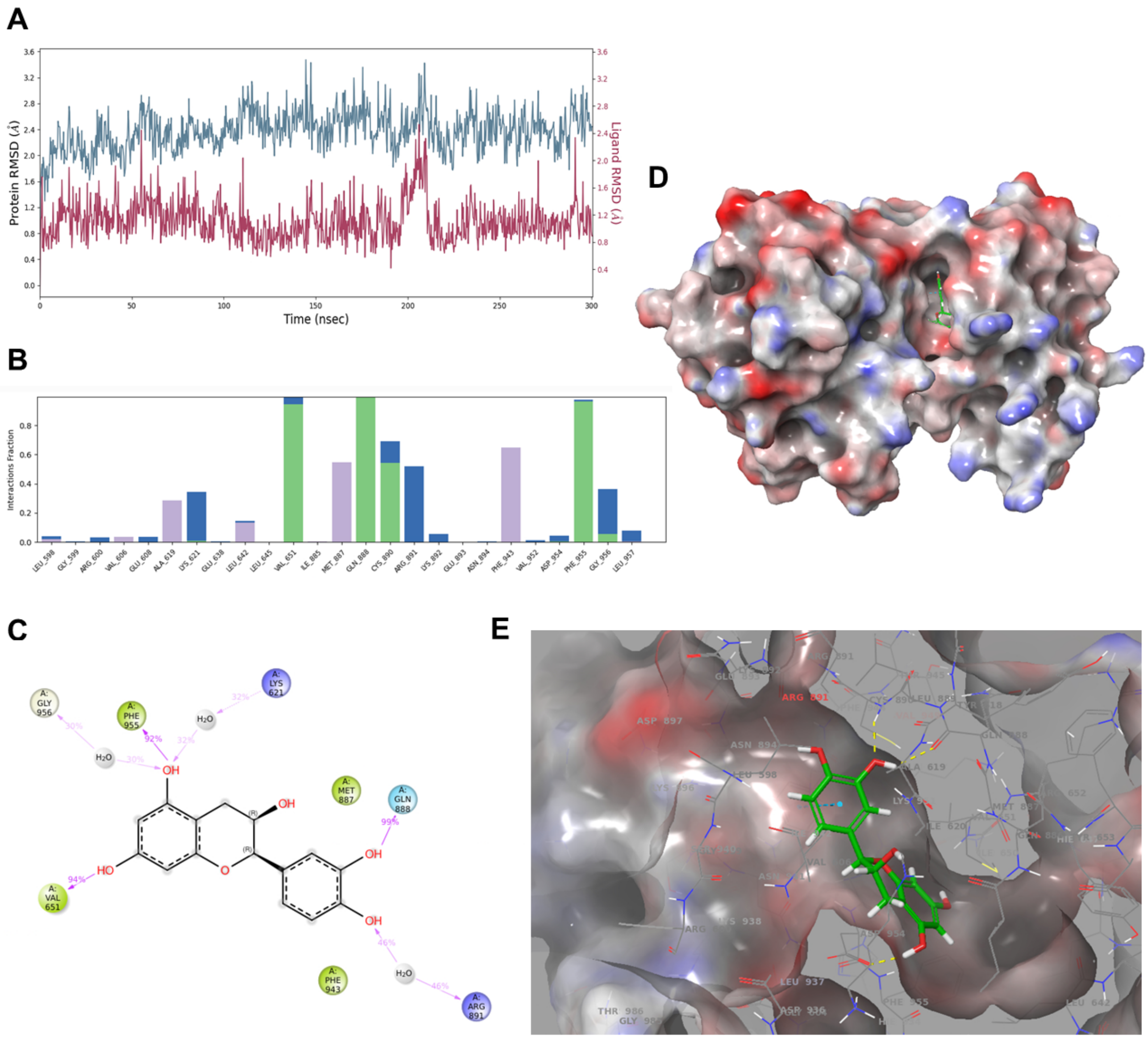

Figure S2. Molecular dynamics between PERK protein and the compound Epicatechin. A) RMSD values for the protein-ligand complex (compound); **B and C)** Occurrence of hydrophilic or hydrophobic interactions, with certain residues, during the dynamics simulation; **D and E)** Three-dimensional demonstration of the interactions between the compound and the protein binding site

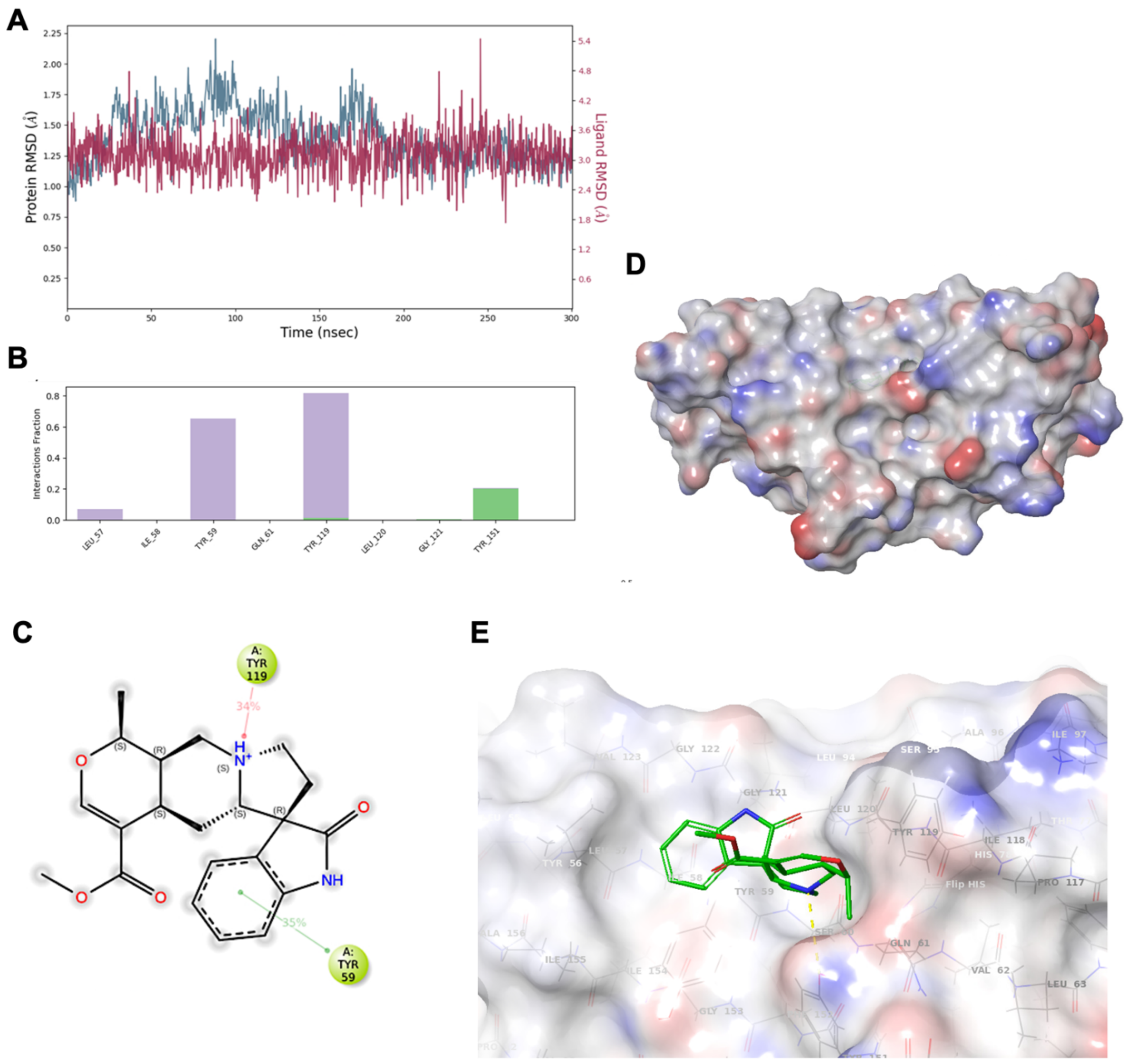

Figure S3. Molecular dynamics between TNF-α cytokine and the compound Mitraphylline. A) RMSD values for the protein-ligand complex (compound); **B and C)** Occurrence of hydrophilic or hydrophobic interactions, with certain residues, during the dynamics simulation; **D and E)** Three-dimensional demonstration of the interactions between the compound and the protein binding site

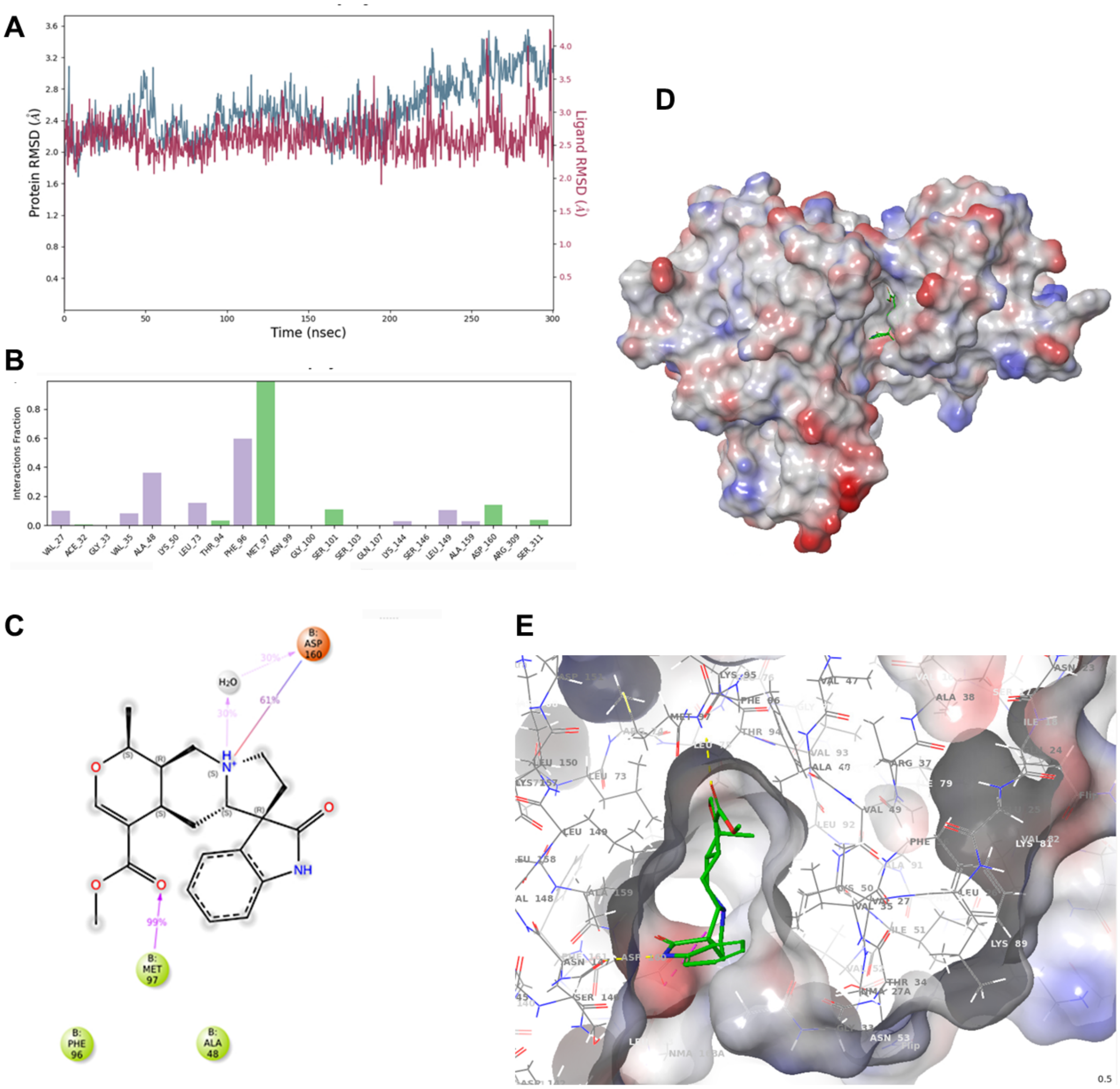

Figure S4. Molecular dynamics between TRAF2 protein and the compound Mitraphylline. A) RMSD values for the protein-ligand complex (compound); **B and C)** Occurrence of hydrophilic or hydrophobic interactions, with certain residues, during the dynamics simulation; **D and E)** Three-dimensional demonstration of the interactions between the compound and the protein binding site

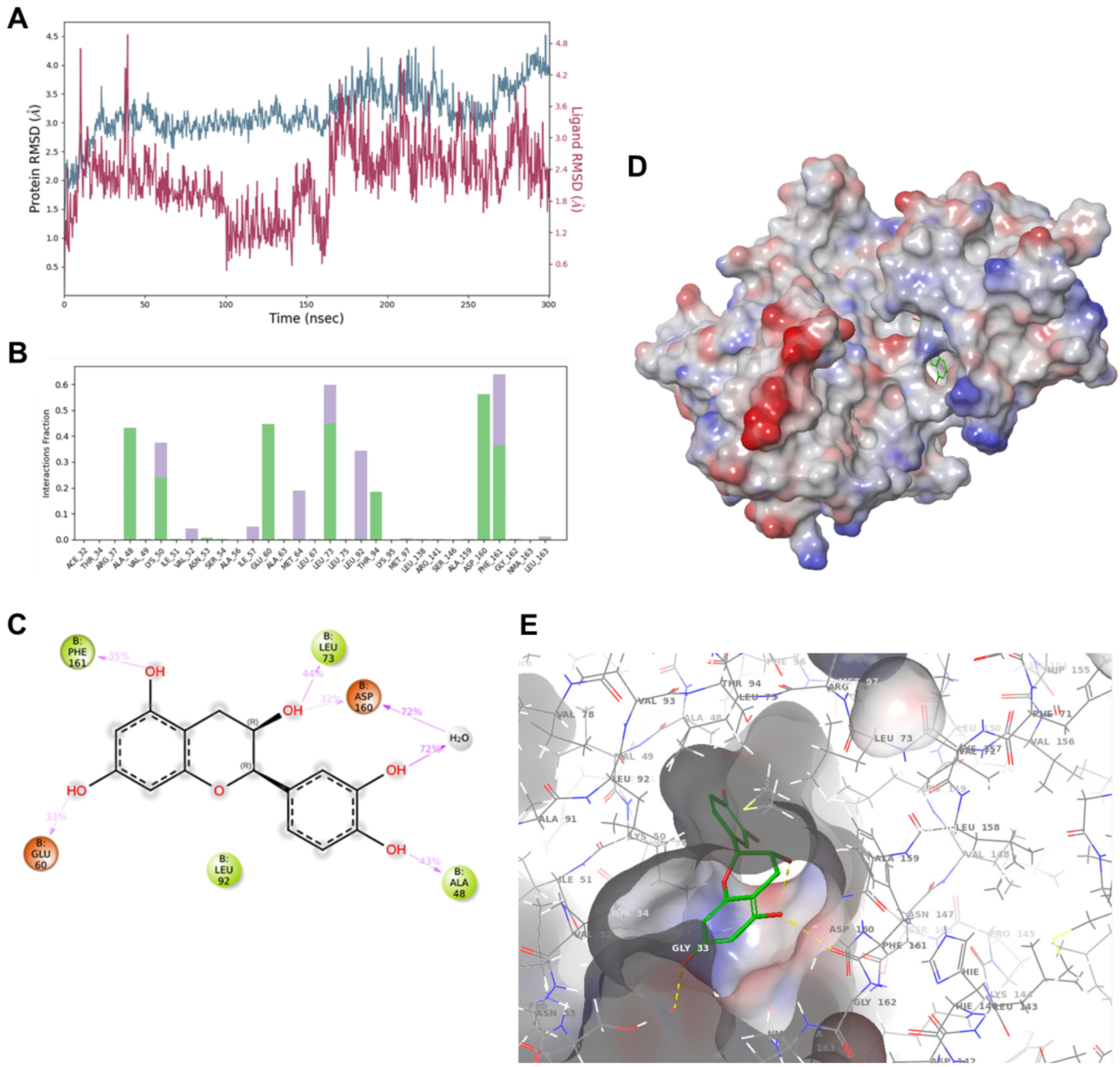

Figure S5. Molecular dynamics between TRAF2 protein and the compound Epicatechin. A) RMSD values for the protein-ligand complex (compound); **B and C)** Occurrence of hydrophilic or hydrophobic interactions, with certain residues, during the dynamics simulation; **D and E)** Three-dimensional demonstration of the interactions between the compound and the protein binding site

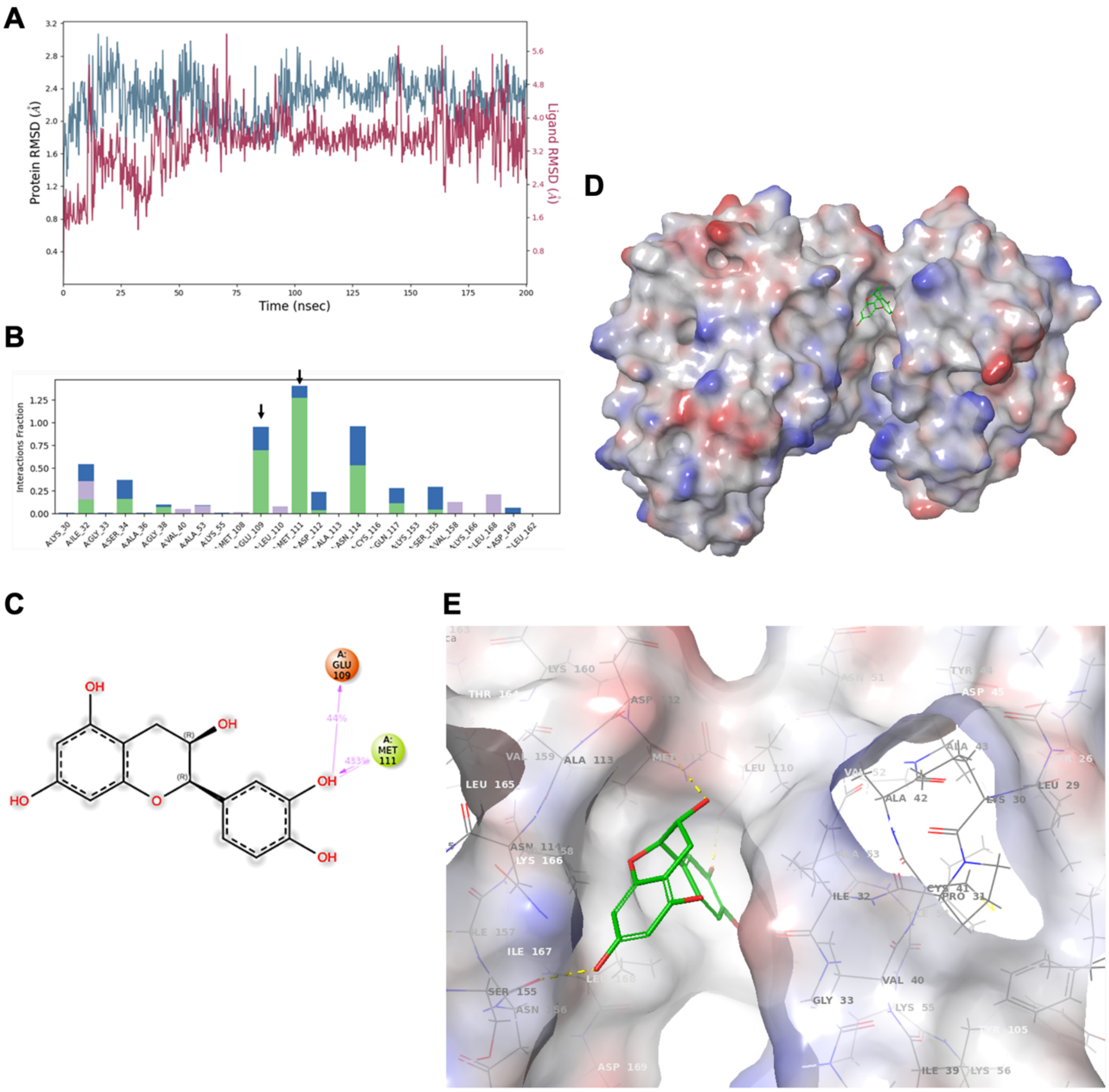

Figure S6. Molecular dynamics between JNK protein and the compound Epicatechin. A) RMSD values for the protein-ligand complex (compound); **B and C)** Occurrence of hydrophilic or hydrophobic interactions, with certain residues, during the dynamics simulation; **D and E)** Three-dimensional demonstration of the interactions between the compound and the protein binding site

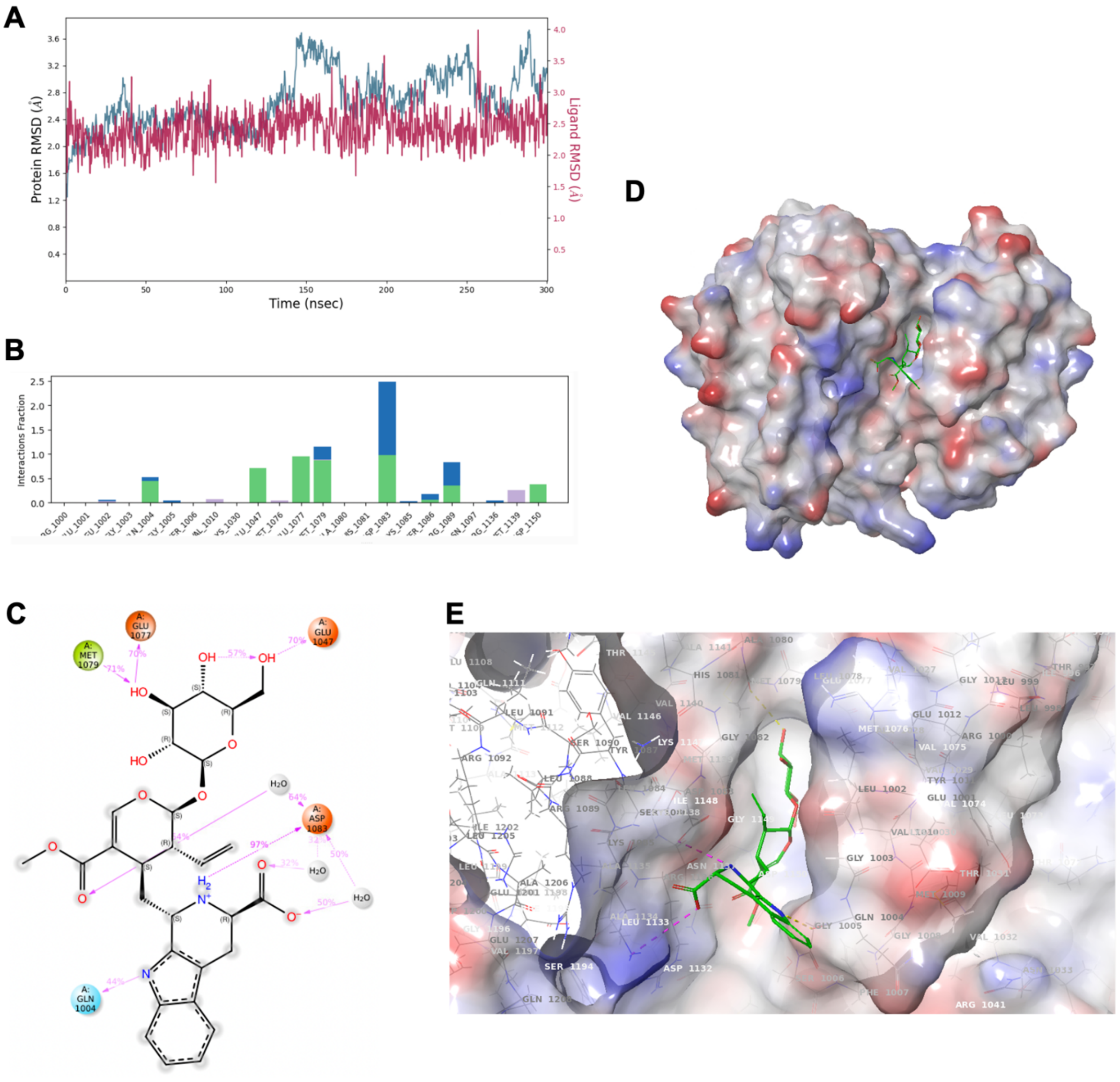

Figure S7. Molecular dynamics between IRS-1 protein and the compound 5-Carboxystrictosidine. A) RMSD values for the protein-ligand complex (compound); **B and C)** Occurrence of hydrophilic or hydrophobic interactions, with certain residues, during the dynamics simulation; **D and E)** Three-dimensional demonstration of the interactions between the compound and the protein binding site

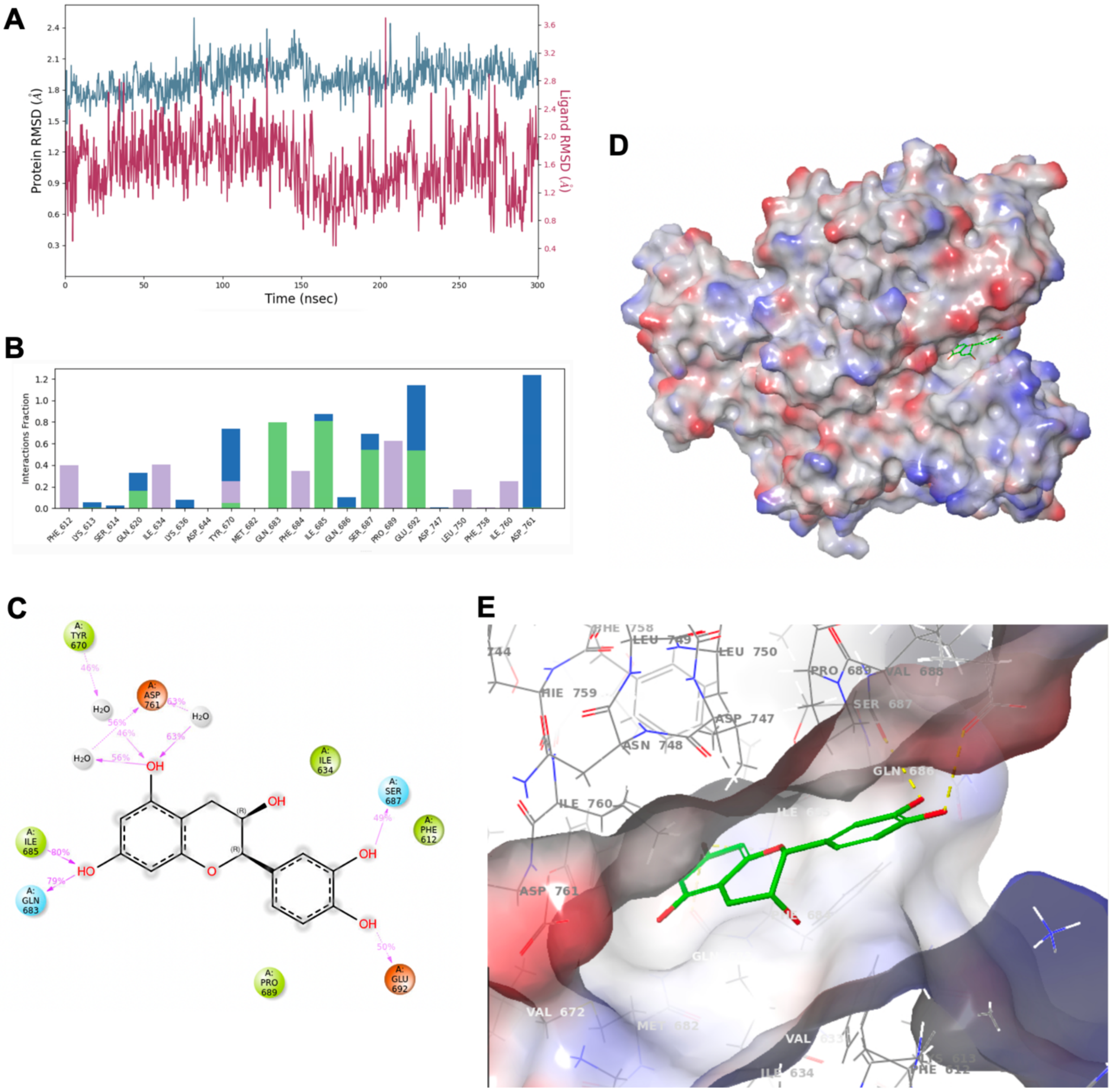

Figure S8. Molecular dynamics between PI3K protein and the compound Epicatechin. A) RMSD values for the protein-ligand complex (compound); **B and C)** Occurrence of hydrophilic or hydrophobic interactions, with certain residues, during the dynamics simulation; **D and E)** Three-dimensional demonstration of the interactions between the compound and the protein binding site

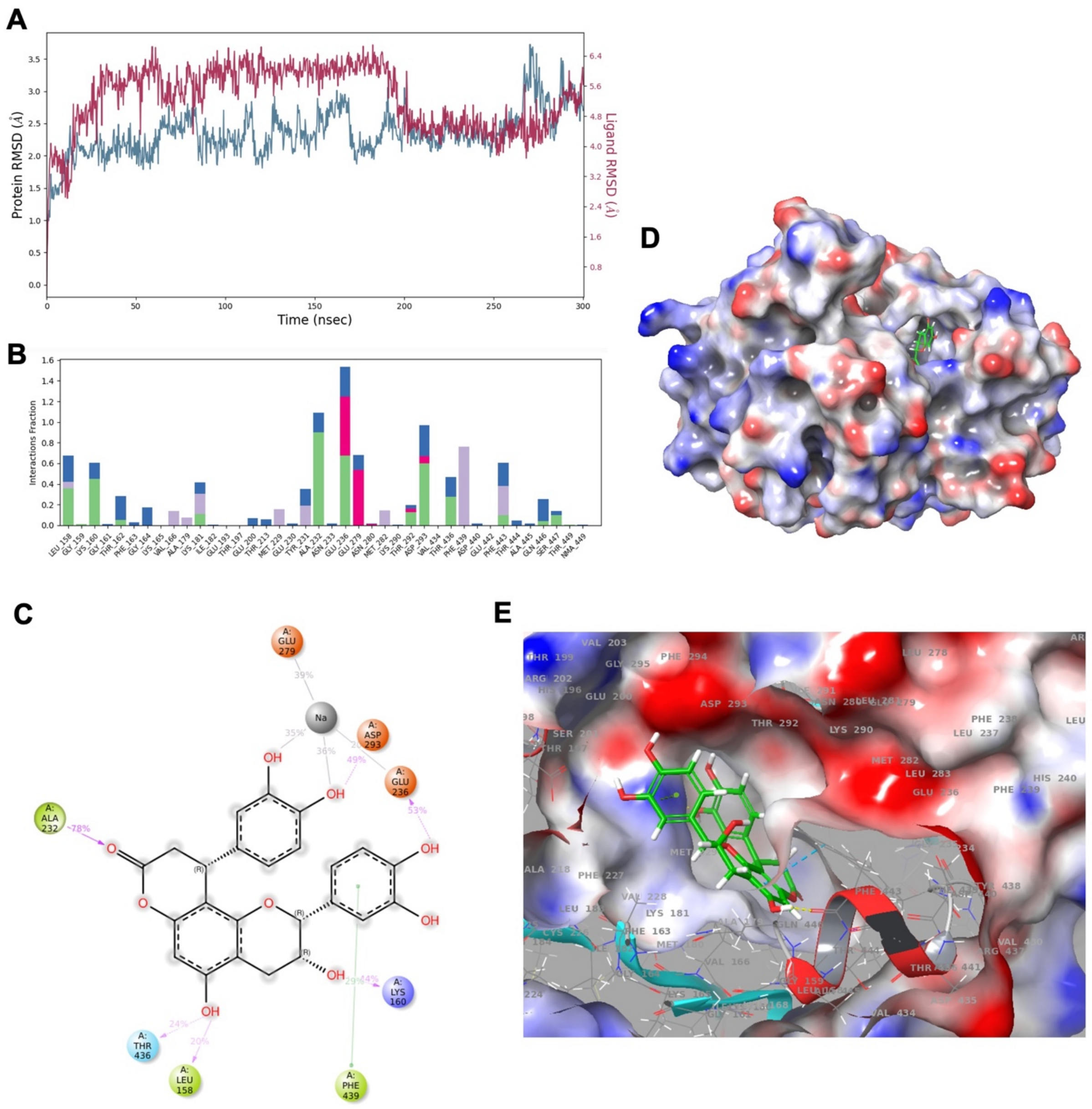

Figure S9. Molecular dynamics between AKT protein and the compound Cinchonain. A) RMSD values for the protein-ligand complex (compound); **B and C)** Occurrence of hydrophilic or hydrophobic interactions, with certain residues, during the dynamics simulation; **D and E)** Three-dimensional demonstration of the interactions between the compound and the protein binding site

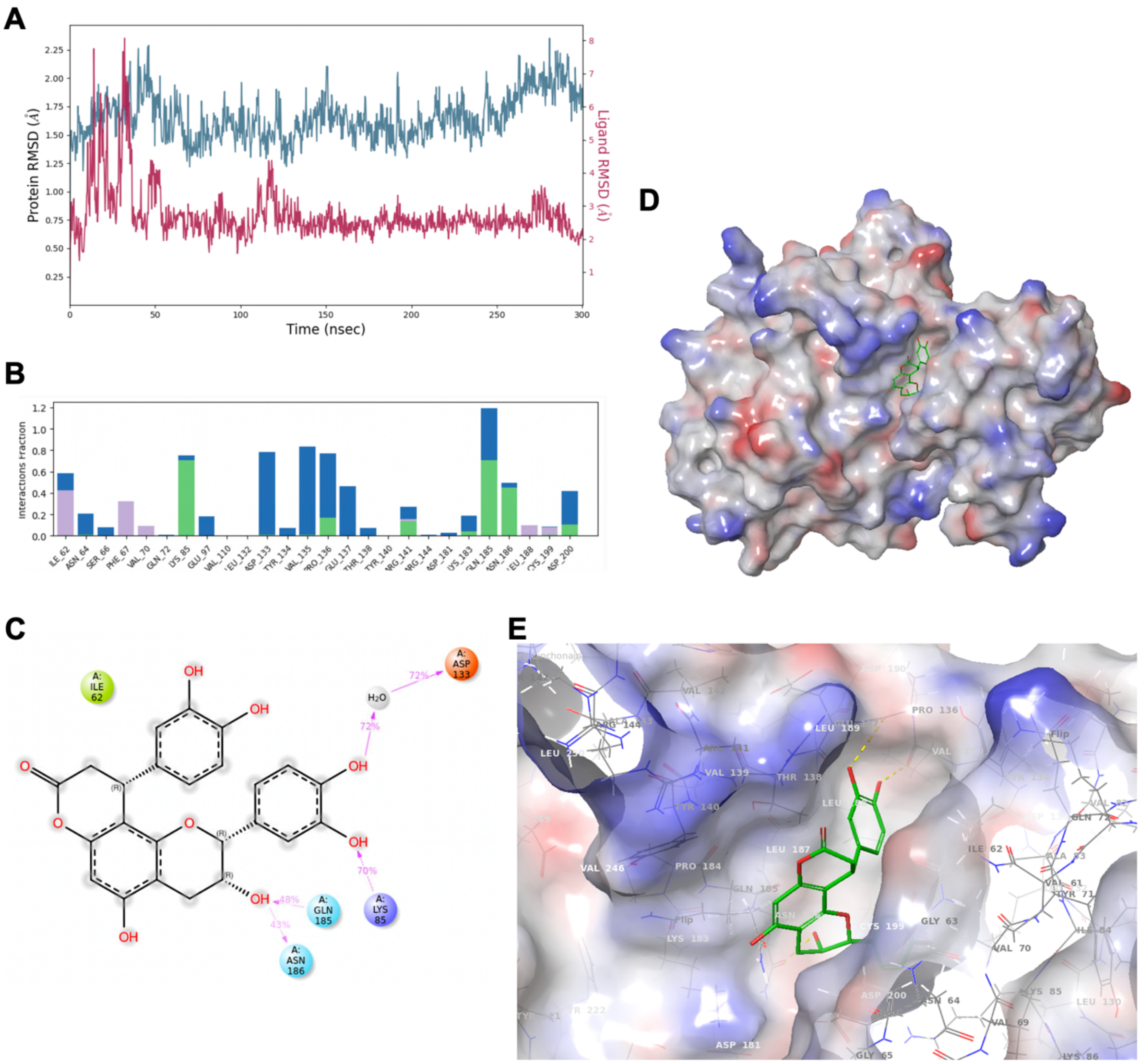

Figure S10. Molecular dynamics between GSK3β protein and the compound Cinchonain. A) RMSD values for the protein-ligand complex (compound); **B and C)** Occurrence of hydrophilic or hydrophobic interactions, with certain residues, during the dynamics simulation; **D and E)** Three-dimensional demonstration of the interactions between the compound and the protein binding site

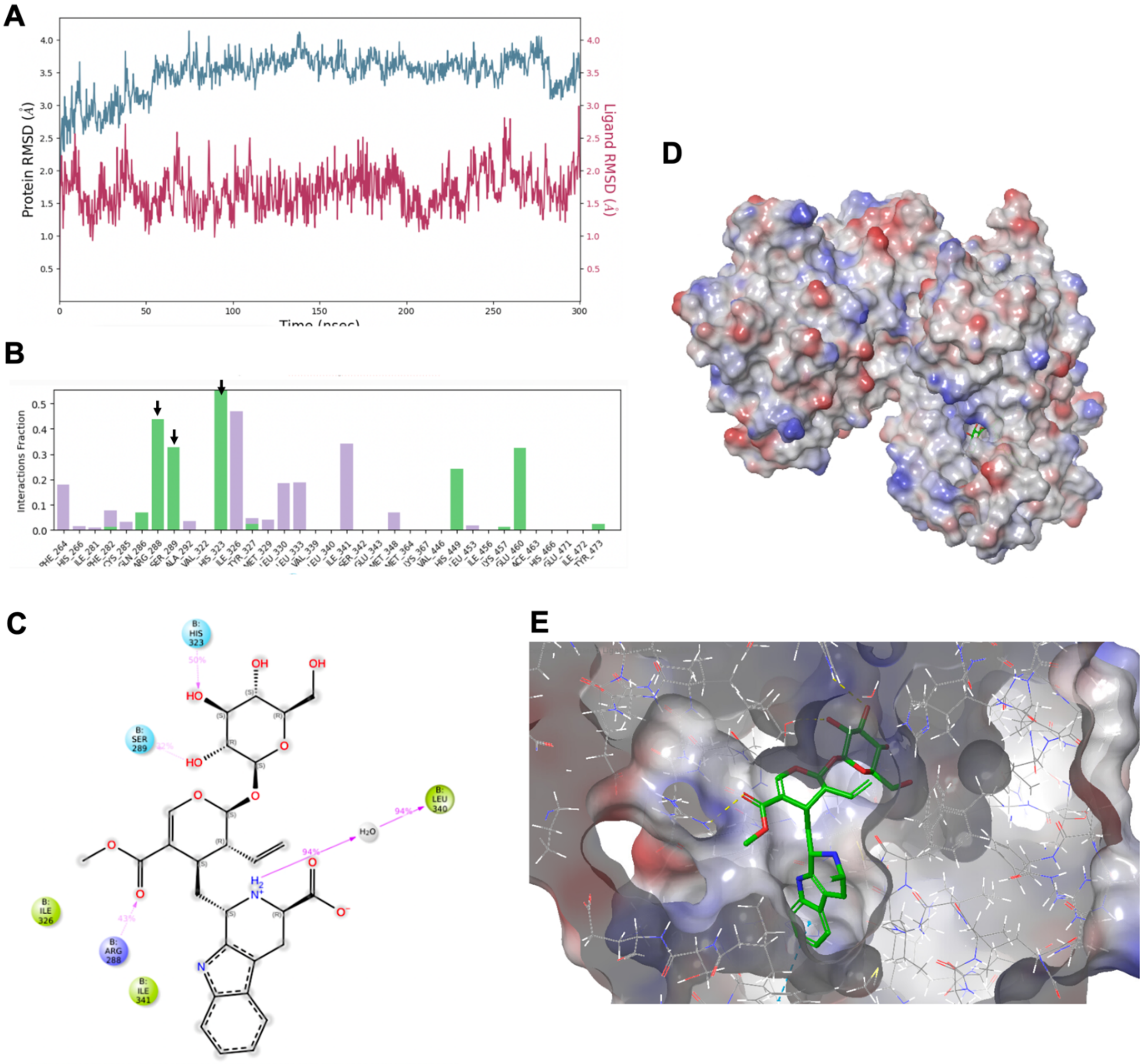

Figure S11. Molecular dynamics between PPARγ protein and the compound 5-Carboxystrictosidine. A) RMSD values for the protein-ligand complex (compound); **B and C)** Occurrence of hydrophilic or hydrophobic interactions, with certain residues, during the dynamics simulation; **D and E)** Three-dimensional demonstration of the interactions between the compound and the protein binding site
